# Ionic Liquids and Water: Hydrophobicity vs. Hydrophilicity

**DOI:** 10.3390/molecules26237159

**Published:** 2021-11-26

**Authors:** Rita F. Rodrigues, Adilson A. Freitas, José N. Canongia Lopes, Karina Shimizu

**Affiliations:** Centro de Química Estrutural, Instituto Superior Técnico, Universidade de Lisboa, 1049-001 Lisbon, Portugal; rita.rodrigues@tecnico.ulisboa.pt (R.F.R.); jnlopes@tecnico.ulisboa.pt (J.N.C.L.)

**Keywords:** ionic liquids, hydrophobicity, hydrophilicity, molecular dynamics, aqueous solutions

## Abstract

Many chemical processes rely extensively on organic solvents posing safety and environmental concerns. For a successful transfer of some of those chemical processes and reactions to aqueous media, agents acting as solubilizers, or phase-modifiers, are of central importance. In the present work, the structure of aqueous solutions of several ionic liquid systems capable of forming multiple solubilizing environments were modeled by molecular dynamics simulations. The effect of small aliphatic chains on solutions of hydrophobic 1-alkyl-3-methylimidazolium bis(trifluoromethyl)sulfonylimide ionic liquids (with alkyl = propyl [C_3_C_1_im][NTf_2_], butyl [C_4_C_1_im][NTf_2_] and isobutyl [iC_4_C_1_im][NTf_2_]) are covered first. Next, we focus on the interactions of sulphonate- and carboxylate-based anions with different hydrogenated and perfluorinated alkyl side chains in solutions of [C_2_C_1_im][C*_n_*F_2*n*+1_SO_3_], [C_2_C_1_im][C*_n_*H_2*n*+1_SO_3_], [C_2_C_1_im][CF_3_CO_2_] and [C_2_C_1_im][CH_3_CO_2_] (*n* = 1, 4, 8). The last system considered is an ionic liquid completely miscible with water that combines the cation N-methyl-N,N,N-tris(2-hydroxyethyl)ammonium [N_1 2OH 2OH 2OH_]^+^, with high hydrogen-bonding capability, and the hydrophobic anion [NTf_2_]^–^. The interplay between short- and long-range interactions, clustering of alkyl and perfluoroalkyl tails, and hydrogen bonding enables a wealth of possibilities in tailoring an ionic liquid solution according to the needs.

## 1. Introduction

Ionic liquids (ILs) are salt-like ionic materials with unusually low melting point temperatures (100 °C being the usually accepted threshold) [1]. It is recognized that this situation is due to (a) low molecular symmetry of the constituting ions, (b) charge delocalization within the ions via resonance or other effects, and (c) the presence of nonpolar moieties such as aliphatic side chains [2]. ILs are being used in a wide range of technological applications, from media in chemical synthesis to components of fuel cells [3,4,5,6,7,8,9,10]. Fine tuning of their chemical properties has been determinant to their success and computational chemistry has played an important part in that process [11,12,13].

Presently, ionic liquids are regarded as nano-segregated fluids and materials. Although they are incapable of forming long-range ordered structures at moderate temperatures (in some cases, even crystallization at lower temperatures is difficult and those ILs tend to undergo glass transitions), they tend to form intermediate-range structures (aggregates) [14]. Such structures are derived, on one hand, by local electroneutrality conditions (a polar 3D network of alternating cations and anions) and, on the other hand, by the segregation between polar and nonpolar regions [15]. A significant contributing factor to the segregation phenomena experienced in many ILs is the presence of alkyl side chains that act as low-charge-density molecular residues [16].

IL–solute interactions have been rationalized considering three specific areas of the IL: the nonpolar regions interact preferentially with nonpolar solutes, such as alkanes via London dispersion forces; the polar network (especially the anions) interacts with water via hydrogen bonding [17]; and, finally, the interface between the polar and nonpolar regions interacts with small dipolar solutes, such as acetone or halogenated hydrocarbons [2]. A summary of different types of IL–solute interactions and their impact in terms of mutual solubility of the corresponding systems is given in the following paragraphs.

The mutual solubilities of ILs and aromatic molecules—benzene [18], hexafluorobenzene derivatives [18,19,20], pyridine, and nicotine [21]—are correlated to the dipolar and quadrupolar moments of those molecules, leading to charge-induced structuration of the IL ions around them [18,19]. For instance, the diverse dipole and quadrupole moments of benzene and its 12 fluorinated derivatives have been conclusively correlated to their solubility in the ionic liquid 1-ethyl-3-methyl-imidazolium bis(trifluoromethanesulfonyl)imide [20], [C_2_C_1_im][NTf_2_]. In the particular case of pyridine, there is complete miscibility in all proportions with some ionic liquids, making it generally more soluble than benzene. This is due to both the presence of a heteroatom in the aromatic ring, which confers a sizable electric dipole moment, and a smaller nonpolar-to-polar domain ratio [21].

In diluted or water-rich solutions, it has been found that water molecules have the two-fold ability of solvating both the anion via hydrogen bonding but also aromatic rings present in some cations. An increase in the length of the alkyl chain of the latter leads to reduced water solubility [22,23]. Moreover, the nature of the head group of the IL also affects the solubility: aromatic cations exhibit higher solubilities than their aliphatic counterparts, e.g., imidazolium- and pyridinium-based ILs have larger water solubilities than their pyrrolidinium- and piperidinium-based counterparts [24]. With regard to the anion, fluorination generally restricts the IL–water solubility windows: solubility in 1-ethyl-3-methylimidazolium tris(pentafluoroethyl)trifluorophosphate, [C_2_C_1_im][FAP], is more limited [25] than in [C_2_C_1_im][PF_6_]. This can be offset by H-bond-type interactions with other interactive center within the anions—e.g., the sulphur atom in the sulfonate and the carbon atom of the acetate ions—that promote the formation of small water/anion alternated clusters [26]. The cation–anion interaction is also a determinant in the behavior of ILs in water. In the case of amino-acid anions, the amino group has a weak interaction with the cation, while the carboxylate group is an excellent hydrogen bond acceptor leading to the breakage of the polar network into smaller fragments/aggregates upon dissolution [27]. Another example is the cholinium cation, where the charge is located on the nitrogen atom and four adjoining methyl/methylene groups. The addition of more OH groups to the cation, and subsequent increase in H-bonding interacting sites, leads to stronger interactions with water, the disruption of the polar network, and much larger miscibility windows with the formation of smaller solvated ionic clusters [28].

The addition of alcohol to IL solutions also highlights the dual role of the alkyl chain of the alcohol molecules, interacting with the polar and nonpolar regions of the IL [29,30]. The water solubility along an IL series is, as expected, lower for ILs with longer alkyl side chains in the cation. However, in IL–octanol solutions, this trend is reversed—there are still interactions between the OH group of the alcohol and the polar part of the ions, but the rest of the octyl chain has to be accommodated in the nonpolar domains of the IL [29].

For smaller alcohol alkyl chains, there is a tendency for self-clustering, with percolation of the simulation box by these aggregates with their accommodation throughout the IL due to the influence of the OH groups, an effect that is lost in longer alcohols [30]. Introducing atoms with lone pairs of electrons capable of accepting a proton and establishing a hydrogen bond with amino-based alcohols adds further possible combinations in which interaction is possible with the IL [31]. As for ethers, it is one of the few systems with ILs that exhibits phase diagrams characterized by the existence of a lower critical solution temperature—a phenomenon that is related to the breaking-up of the hydrogen-bonded network that can be formed between the functionalized cation of the ionic liquid and the oxygen atom of the ether molecule [32].

As previously discussed, nonpolar solutes such as alkanes have a higher affinity for the nonpolar regions of the IL. In bromo- and chloro-monosubstituted alkanes, ILs show higher solubility towards shorter alkyl-side chains and bromide-based solutes, as opposed to the chloride-based, but there is still a measure of interaction between the halogens and the hydrogen atoms of the imidazolium ring and the substituted carbon of the haloalkane and the oxygen atoms of the anions with shorter chains [33]. Xenon can be viewed from a similar lens, modeled with similar potential parameters to the n-alkanes, xenon will start to move away from the vicinity of the imidazolium with the progressive increase in the alkyl side chain in the IL [34]. The solubilities of *n*-butane and 2-methylpropane [35] confirm all the previously described issues—the more flexible and less bulky n-butane is well accommodated in the nonpolar domains of ILs (especially those with linear side chains), whereas the bulkier isobutane solute is not accommodated so efficiently in such ILs.

Hydrotropes are a class of compounds that can enhance the solubility of hydrophobic substances in aqueous media. The unique solvation properties of ILs can be used to that effect, with the solubilization of solutes that are sparingly soluble in pure water due to the formation of solute–IL and water–IL aggregates [36]. Using molecular simulation, coaggregates between the hydrophobic solute and the IL ions in aqueous solution have been validated. In the case of IL aqueous solutions containing vanillin, the hydrotrope effect was achieved, with interactions between specific vanillin–cation, cation–anion, and anion–water identified. For ibuprofen, the interactions with ILs, although present, are mostly restricted to a network formed by the cations and ibuprofen at the surface of the later subphase [37].

SILs (solvated ionic liquids) are a subclass of ILs, consisting of a metal cation bound to a stoichiometric quantity of coordinating ligands via strong Lewis acid–base interactions that yield stable complex cations and counter ions in the bulk liquid. Strong complexation between Li^+^ and glyme molecules produces stable [Li(glyme)] cations (i.e., a good SIL). Conversely, a “poor” SIL is characterized by stronger interactions between Li^+^ and the anion, which promotes the presence of uncoordinated glyme in solution [38]. Thus, different counter ions yield different types of SIL: in [Li(G4)][NO_3_], strong Li–anion interactions leads to Li-rich and Li-depleted regions in the bulk; conversely, in [Li(G4)][NTf_2_], strong Li–glyme interactions generate stable complex cations, with well solvated and uniformly distributed Li cations in the bulk [39]; in [Li(G4)][NTf_2_], Li–anion connections are weaker than in [Li(G4)][NO_3_], with the anion generally exhibiting two monodentate coordinations to Li^+^ [40].

LCILs (liquid crystal ionic liquids) are another IL subclass that exhibits liquid crystalline meso-phases at moderate temperatures. The unique solvent properties of LCILs, namely their ordered structures, can influence the stereochemical outcome of a Diels–Alder (DA) reaction between cyclopentadiene and methyl acrylate. This is due to the different hydrogen bonding interactions between the LCIL reaction media and the exo- or endo-transition states in solvents with layered (smectic, SmA) ordering—it restricts the accessibility of hydrogen bond donors of the IL cation to both DA products—as compared to the isotropic phase. Overall, the effective polarity of the solvent lowers and there is preferential hydrogen bonding to the exo-product in the polar region of the segregated bilayer of the SmA phase of the LCILs [41].

Finally, the bulk properties of ILs can be manipulated by using mixtures of different ILs at differing proportions and this can lead to the tailoring of the phase: smaller aggregates in the matrix achieving both nonpolar domains and a bicontinuous network [42], changing the affinity towards water [43], introducing small clusters while still maintaining percolation [44], and even bridging between different aggregates [45].

**Chart 1:**
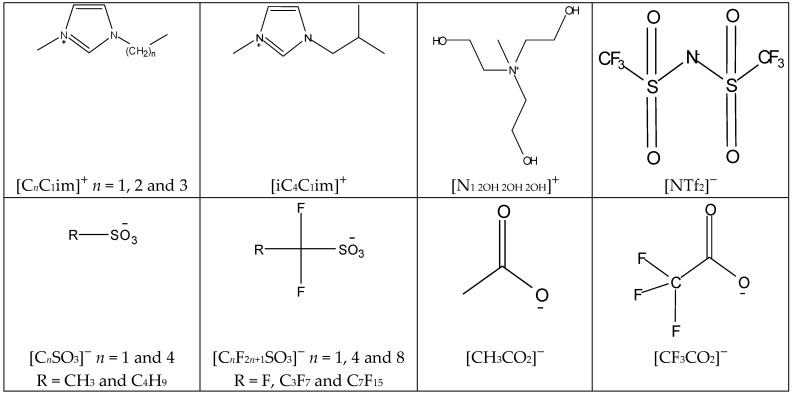
Molecular structure and acronyms of the ions composing the ionic liquids studied in this work.

The different systems modeled and simulated in the present work will address, in more detail, some of the issues discussed in the previous paragraphs. The focus will be on aqueous solutions of diverse ionic liquids (Chart 1), namely those containing (a) different cations with small alkyl sidechains (including branched ones); (b) different anions based on sulfonate or carboxylate moieties attached to hydrogenated or fluorinated alkyl chains; and (c) cations with high hydrogen-bonding capabilities.

## 2. Results and Discussion

### 2.1. Aqueous Solutions of [C_3_C_1_im][NTf_2_], [C_4_C_1_im][NTf_2_], and [iC_4_C_1_im][NTf_2_]

The three IL systems studied in this section are known to be hydrophobic: none of them are completely miscible with water at room temperature. This means that we will address structural issues related to the addition of modest amounts of water to the pure ILs (below the corresponding saturation limits). To facilitate comparisons between systems, a common value of ionic liquid mole fraction, X_IL_ = 0.70 (wt_IL_ = 98%), for the IL-rich aqueous IL solutions was established (please refer to Table 1 for weight and mole fractions of all systems studied). In other words, this mole fraction corresponds to ILs that contain small amounts of water and the amount of water is at the saturation limit. The solubility of the ILs in pure water is very low and the corresponding water-rich IL aqueous solutions were not considered. All systems have a common anion combined with three cations with rather small alkyl side chains. The objective of this section is to determine how small changes in those side chains (increase in just one –CH_2_– group; linear versus branched chains) affects the uptake of water in the IL aqueous solutions and possible structural differences.

**Figure 1:**
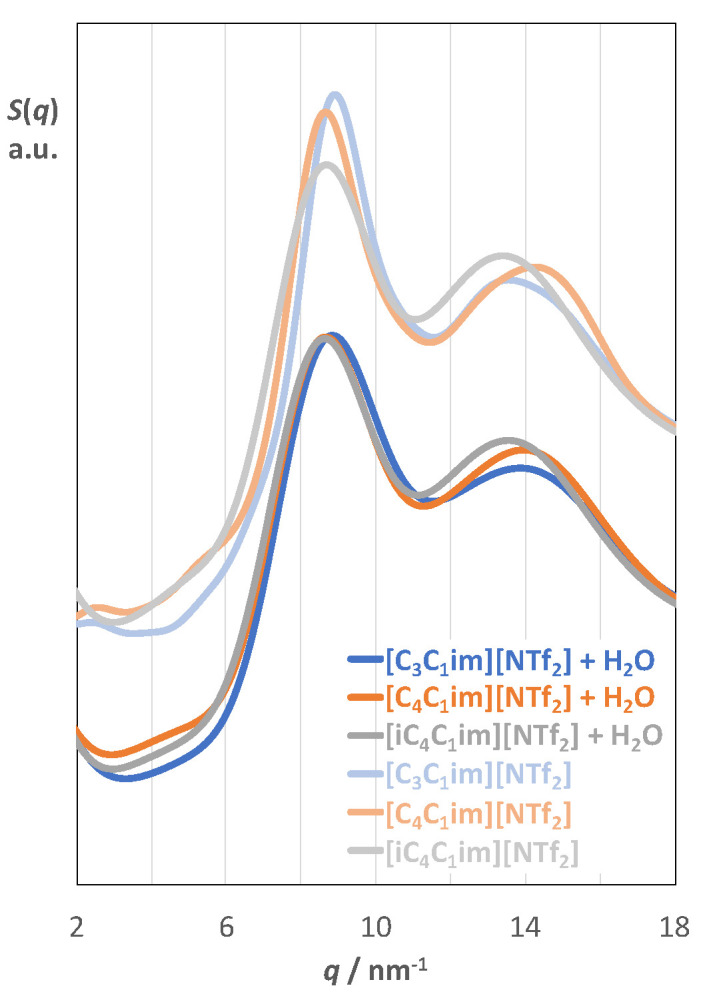
Structure factor functions, *S*(*q*), calculated at 320 K for pure [C_3_C_1_im][NTf_2_], [C_4_C_1_im][NTf_2_], and [iC_4_C_1_im][NTf_2_] ILs and its aqueous solutions with X_IL_ = 0.70 (wt_IL_ = 98%).

Figure 1 shows the structure factor functions, *S*(*q*), for [C_3_C_1_im][NTf_2_], [C_4_C_1_im][NTf_2_], and [iC_4_C_1_im][NTf_2_] as pure substances and as aqueous solutions with a water mole fraction, X_IL_ = 0.70. All *S*(*q*) functions are quite similar, with two prominent peaks at around 9 and 14 nm^−1^, corresponding to charge ordering peaks (COPs) and contact peaks (CPs) of the different liquids. The COP reflects the existence of characteristic distances between ions within the polar network of the IL; the CP reflects characteristic contact distances between atoms of different ions and molecules. The COPs are distinctive features of ionic liquids that are generally absent in the *S*(*q*) functions of (neutral) molecular fluids. Because there are almost no changes in the peak positions of the *S*(*q*) functions between the pure ILs and their aqueous solution counterparts, one can conclude that the water molecules present in solution do not interfere with the overall structure of the ILs and can occupy spaces in the vicinity of the cations and anions without disrupting their charge ordering. In other words, water–ion interactions are insufficient to split the cohesion of the cation–anion polar network at low water fractions [46,47]. The COP peak for [C_3_C_1_im][NTf_2_] has a *q* value 8.9 nm^−1^, slightly larger than the other two ILs that have an additional CH_2_ unit at the alkyl chain. Such *q* value corresponds to characteristic distances in the polar network of *d* = 2π/*q* = 0.704 nm, the distance between the centers of charge of two anions or cations separated by a common counter-ion. The slightly lower *q* values for [C_3_C_1_im][NTf_2_] and [iC_4_C_1_im][NTf_2_] (corresponding to smaller *d* values) reflect a less efficient packing of the charged parts of the ions within the polar network caused by the slightly bulkier alkyl side chains in the cations. Hence, [C_3_C_1_im][NTf_2_] is expected to have a more compact polar network, with both [C_4_C_1_im][NTf_2_] and [iC_4_C_1_im][NTf_2_], being comparatively more stretched.

There is a small shoulder at approximately 4 nm^−1^ for [C_4_C_1_im][NTf_2_], both in the pure IL and in the aqueous solution. This corresponds to the emergence of a PNPP (polar–nonpolar peak) that reflects characteristic distances between strands of the polar network separated by the (nonpolar) alkyl moieties of the cations. The shoulder is more subdued for [iC_4_C_1_im][NTf_2_] and suppressed for [C_3_C_1_im][NTf_2_]. PNPPs are known to occur only after a given threshold value for the length of the alkyl side chains present in the ions. Kurnia et al. [24] have previously reported that the spacing between polar strands mediated by alkyl side chains is slightly larger for [C_4_C_1_im][NTf_2_] than for its branched counterpart, [iC_4_C_1_im][NTf_2_].

**Figure 2:**
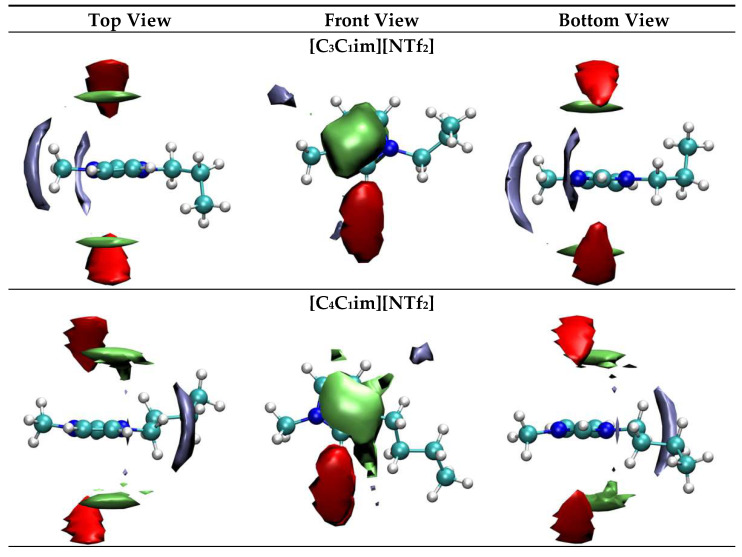

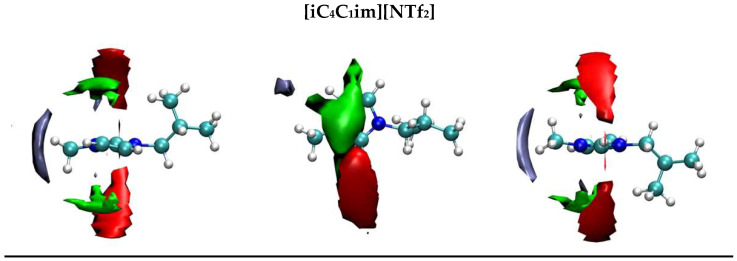
Selected spatial distribution functions around the imidazolium cation in aqueous mixtures X_IL_ = 0.70 (wt_IL_ = 98%) of [C_3_C_1_im][NTf_2_], [C_4_C_1_im][NTf_2_], and [iC_4_C_1_im][NTf_2_] at 320 K. The red and green colors represent N and F atoms of the anion, respectively, and light blue depicts water O atom. The isosurface value is 70% of the maximum number density.

Figure 2 shows different spatial distribution functions (SDFs) around the cation for aqueous solutions of [C_3_C_1_im][NTf_2_], [C_4_C_1_im][NTf_2_], and [iC_4_C_1_im][NTf_2_]. The [NTf_2_]^−^ anion can surround the charged moiety of the cation (the imidazolium ring), positioning the CF_3_ groups above and below the plane of the ring and the SO_2_ groups mainly near the acidic hydrogen at C2 (the green contours in the SDFs represent the position of the nitrogen atom of the anion, as a proxy for the position of the sulfonate groups). On one hand, such positioning reflects the basicity of the SO_2_ groups (and their affinity for the more acidic hydrogens of the cation) and, on the other hand, the possibility of fluorine–aromatic (F-π) interactions. This situation hinders the approximation of water molecules at those positions (C2 position, above and below the aromatic plane). This means that water molecules will be found mainly near the other less acidic hydrogens (C4 and C5 positions), with little disturbance on the size of the polar network, and as shown also in Figure 2. Interestingly, the water molecules slightly prefer the C4 position in [C_3_C_1_im][NTf_2_] and [iC_4_C_1_im][NTf_2_] and the C5 position in [C_4_C_1_im][NTf_2_]. The existence of a more structured nonpolar domain due to the existence of longer (C4) chains (as hinted at by the PNP shoulder) probably declutters the vicinity of the C5 and can help explain such effect.

**Figure 3:**
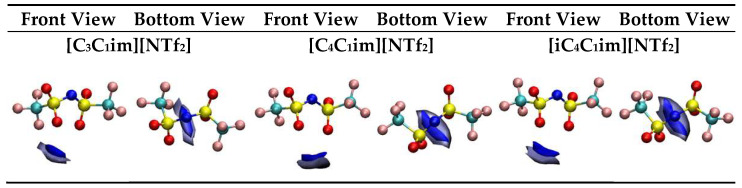
Selected spatial distribution functions around the [NTf_2_]^−^ anion in aqueous mixtures X_IL_ = 0.70 (wt_IL_ = 98%) of [C_3_C_1_im][NTf_2_], [C_4_C_1_im][NTf_2_], and [iC_4_C_1_im][NTf_2_] at 320 K. The blue color represents the H atom of cations attached to the C2 carbon between nitrogen atoms of the imidazolium ring and light blue depicts the water O atom. The isosurface value is 70% of the maximum number density.

The SDFs around the anions are presented in Figure 3 and show that apparently both the water molecules and the C2 hydrogen of the cation tend to interact with the anion at the same position (the charged part of the anion containing the nitrogen atom and the sulfonate groups). This apparent inconsistency (the SDFs of Figure 2 have shown that the water molecules and the anions interact with the cation at different positions) points to the fact that SDFs are sometimes quite challenging to interpret. The difficulty comes from the fact that the contours are the result of averages performed over selected pairs of atoms or molecular sites that can adopt different relative positions within the ions or molecules being considered. In the case of the imidazolium anion, things are particularly complex because the interactions are mainly performed via the most electronegative atoms (the four oxygen atoms of the sulfonate groups). Looking closely at those two SO_2_ groups, one can notice that there is an O atom in each group assuming an axial orientation with respect to the S-N-S plane, while the other O atom takes an equatorial orientation. Moreover, the O-O distance between the axial pair is smaller than the equatorial pair. This axial/equatorial orientation of O atoms is present in both *cisoid* and *transoid* conformers and influences the organization of other molecules and ions around the [NTf_2_]^−^ anion. What the SDFs in Figure 3 indicate is simply that the anion interacts with water and the C2 hydrogen of the cation preferentially, with one of the axial O atoms in the SO_2_ groups. The flexibility of the molecule and the existence of multiple O atoms results on the apparently superimposed average contours presented in the SDFs.

**Figure 4:**
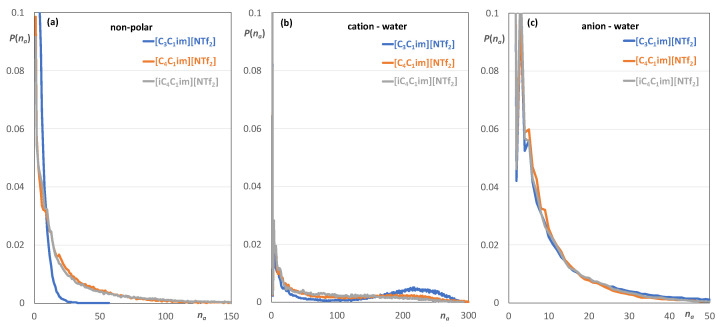
Discrete probability distribution of aggregates in aqueous mixtures X_IL_ = 0.70 (wt_IL_ = 98%) of [C_3_C_1_im][NTf_2_], [C_4_C_1_im][NTf_2_], and [iC_4_C_1_im][NTf_2_]: (**a**) tail–tail; (**b**) cation–water and (**c**) anion–water aggregates.

Figure 4 shows the size distributions for nonpolar, cation-water and anion-water aggregates contained in the aqueous solutions of [C_3_C_1_im][NTf_2_], [C_4_C_1_im][NTf_2_], and [iC_4_C_1_im][NTf_2_] (300 IL pairs in each system). The water-water aggregates are presented in Figure S1 of the Supplementary Materials. The nonpolar aggregates are small in all cases (and smallest for [C_3_C_1_im][NTf_2_], with 21% of isolated alkyl side chains). The addition of small amounts of water induces some nonpolar aggregation on [C_4_C_1_im][NTf_2_] and [iC_4_C_1_im][NTf_2_] (and more segregation from the polar network). In both cases, the nonpolar aggregates are comparatively larger, with just 10% of isolated alkyl side chains and maximum aggregate sizes of 202 for [C_4_C_1_im][NTf_2_] and 234 for [iC_4_C_1_im][NTf_2_], in agreement with the existence of incipient PNPP shoulders (Figure 1). The preferential solvation of the cation can be observed comparing cation–water aggregates (center of mass of the imidazolium ring–oxygen atom of water) and anion–water aggregates (nitrogen atom of the anion–oxygen atom of water): cation–water aggregates have size distributions shifted to larger values than anion–water aggregates. Conversely, 26% of cations show absence of water in their vicinity, whereas the number rises to 35% for the anions. Moreover, the size distribution of cation–water aggregates indicates that [C_3_C_1_im][NTf_2_] is more soluble than the other two ILs (larger aggregates with a maximum at rather large values (Figure 4b).

**Figure 5:**
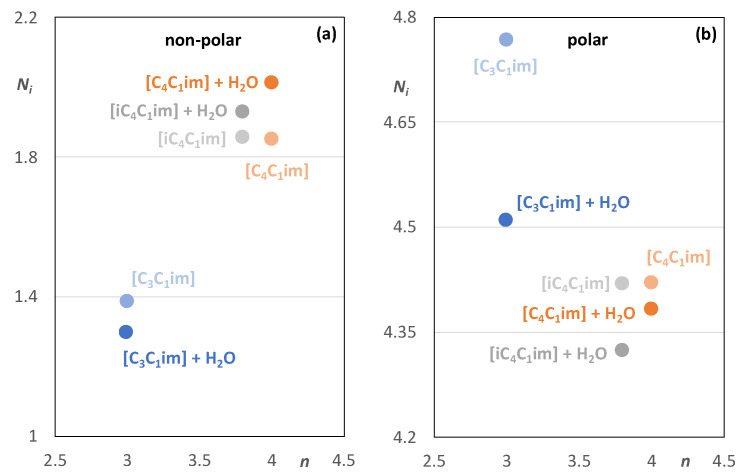
Average number of contact neighbors (*N_i_*) for pure ILs and aqueous mixtures X_IL_ = 0.70 (wt_IL_ = 98%) of [C_3_C_1_im][NTf_2_], [C_4_C_1_im][NTf_2_], and [iC_4_C_1_im][NTf_2_] as a function of the number of carbons in alkyl chain: (**a**) tail–tail and (**b**) cation–anion aggregates.

**Figure 6:**
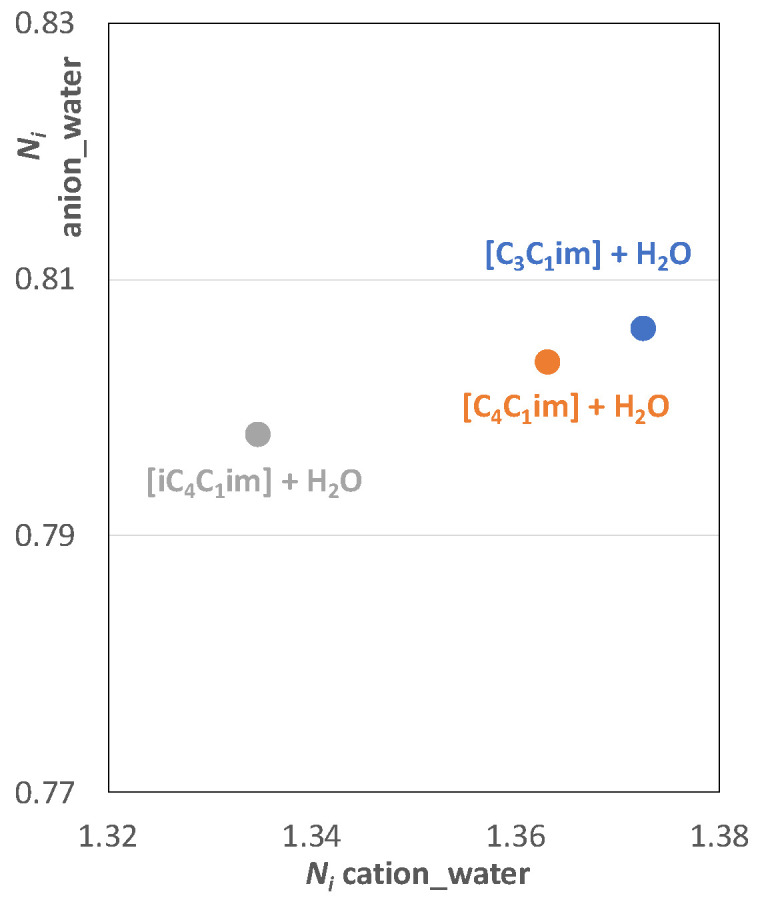
Average number of water neighbors around the cation (*N_i_* cation_water) vs. the anion (*N_i_* anion_water) for aqueous mixtures X_IL_ = 0.70 (wt_IL_ = 98%) of [C_3_C_1_im][NTf_2_], [C_4_C_1_im][NTf_2_], and [iC_4_C_1_im][NTf_2_].

The alkyl side chains of pure [C_4_C_1_im][NTf_2_] and [iC_4_C_1_im][NTf_2_] display similar average numbers of contact neighbors (Figure 5a). The inclusion of water slightly increases such average numbers. Pison et al. [35] have reported that linear chains show a better packing when compared to the branched chains, which leads to a higher number of average neighbors, especially in the presence of water. On the other hand, in the case of [C_3_C_1_im][NTf_2_], water addition decreases the number of contact neighbors. This suggests that since the chain is short and not far removed from the polar network, the addition of water molecules in the same region further separates the alkyl side chains, allowing higher mobility of the nonpolar tails. This should be taken into consideration in ionic liquid/water extraction processes in which the nonpolar nanophase plays a role [48]. The average number of contact neighbors within the polar network (contacts between the charged part of an ion and the charged part of its counterion) is presented in Figure 5b. Pure [C_4_C_1_im][NTf_2_] and [iC_4_C_1_im][NTf_2_] again show similar values; however, when water is added, the average number of contact neighbors decreases, an expected outcome since some water molecules are now present, adjacent to the anions and cations. Figure 6 shows the average number of contact neighbors in anion–water aggregates as a function of the corresponding values for cation–water aggregates. The graph shows that there are, on average, more water molecules surrounding cations than anions (70% more) and that ions in [C_3_C_1_im][NTf_2_] are more “hydrated” than in [C_4_C_1_im][NTf_2_] or [iC_4_C_1_im][NTf_2_].

### 2.2. Aqueous Solutions of [C_2_C_1_im][C_n_F_2n+1_SO_3_], [C_2_C_1_im][C_n_H_2n+1_SO_3_], [C_2_C_1_im][CF_3_CO_2_], and [C_2_C_1_im][CH_3_CO_2_]

**Figure 7:**
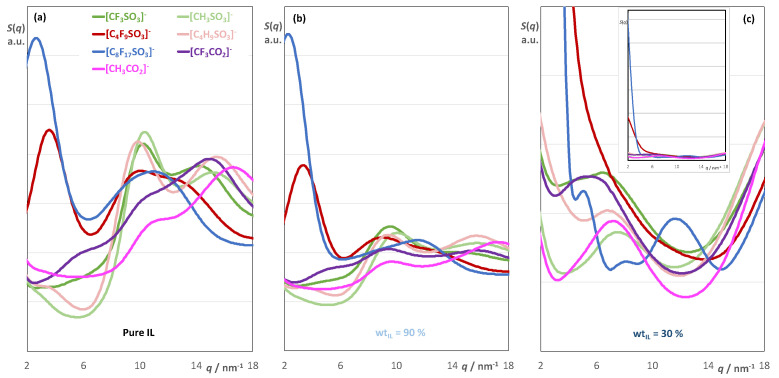
Structure factors calculated at 300 K for (**a**) pure ILs, (**b**) aqueous solutions with wt_IL_ = 90% (X_IL_ = 0.23–0.49), and (**c**) wt_IL_ = 30% (X_IL_ = 0.01–0.04).

In this set of systems, we will focus on the interactions between anions with different hydrogenated and fluorinated alkyl side chains combined with carboxylate or sulfonate headgroups. The aqueous solutions will cover a more extensive range of water composition when compared to the previous group of systems. Due to this fact, concentrations will be given in IL weight fraction, wt_IL_. Figure 7a shows the structure factor functions, *S*(*q*), calculated at 300 K for several pure ILs containing the [C_2_C_1_im]^+^ cation; the respective aqueous solutions with 90% and 30% wt_IL_ are presented in Figure 7b,c (roughly speaking, for these ILs wt_IL_ = 90% represents X_IL_ in 0.23–0.49 range, while wt_IL_ = 30% denotes X_IL_ in 0.01–0.04 range). For the pure ILs, the deconvolution of the two peaks at around 10 and 14 nm^−1^ have been previously assigned [49] to the charge ordering peak (COP) and the contact peak (CP), respectively. As mentioned above, the latter is related to characteristic distances between contacting atoms from different ions, whereas the former is an intrinsic feature of ILs that is related to characteristic distances within the polar network. The pure ILs, [C_2_C_1_im][C_4_F_9_SO_3_] and [C_2_C_1_im][C_8_F_17_SO_3_], also exhibit conspicuous low-*q* peaks around 3 nm^−1^—known as pre-peaks or polar nonpolar peaks (PNPPs)—a consequence of the nano-segregation between the polar and nonpolar domains within the IL [50]. At low water concentration (wt_IL_= 0.9; X_IL_ = 0.23–0.49), the PNPP, COP, and CP positions are similar to the ones found for the pure ILs, indicating that the water molecules can occupy positions adjacent to the polar network of the ILs without promoting the disruption of too many cation–anion interactions. However, the intensity of the PNPPs in imidazolium-based ILs with longer alkyl chains increases significantly in relation to the COPs and CPs (Figure 7b), a possible indication of a stronger segregation between polar and nonpolar domains (the former containing the most water molecules, the latter very few of them).

At higher water concentrations, wt_IL_= 0.3 (X_IL_ = 0.01–0.04, Figure 7c), it is observed that the COPs shift to lower *q* values, a clear indication that the polar region of the IL suffers a broadening/swelling in order to accommodate the water molecules, which naturally prefer a polar environment and avoid the nonpolar segments of the IL. Looking at the inset in Figure 7c, where the intensity of PNPP in ILs with longer chain lengths is more evident, it is clear that, even though the percentage of the IL is low, the pre-peak is still present. As previously reported by Bastos et al. [26], simulated boxes of diluted solutions are not solely described by isolated solvated ions. In fact, higher chain lengths enable the formation of large-size “micelle-like” aggregates in water and even smaller ILs display a certain amount of clustering. In fact, with the exception of [CH_3_CO_2_]^−^ and [CH_3_SO_3_]^−^, all other ILs present some degree of nonpolar segregation at wt_IL_ = 30% (X_IL_ = 0.01–0.04).

**Figure 8:**
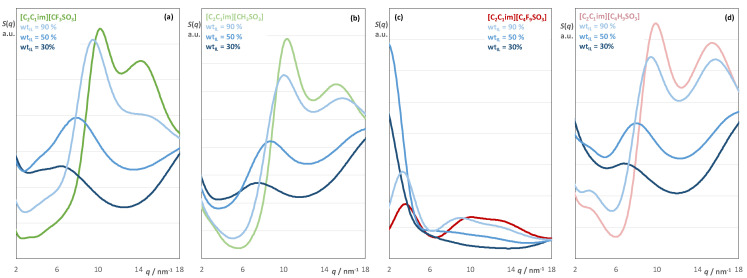
Structure factor for (**a**) [C_2_C_1_im][CF_3_SO_3_]; (**b**) [C_2_C_1_im][CH_3_SO_3_]; (**c**) [C_2_C_1_im][C_4_F_9_SO_3_]; and (**d**) [C_2_C_1_im][C_4_H_9_SO_3_]. Data for pure ILs calculated at 300 K and aqueous solutions with wt_IL_ = 30% (X_IL_ = 0.01–0.04), wt_IL_ = 50% (X_IL_ = 0.05–0.10), and wt_IL_ = 90% (X_IL_ = 0.23–0.49).

In Figure 8, the evolution of the structure factor with increasing addition of water can be observed in higher detail for some ILs with fluorinated anions and their hydrogenated analogues. The shift of the COP peak to lower *q* values upon addition of water in [C_2_C_1_im][CF_3_SO_3_] and [C_2_C_1_im][CH_3_SO_3_] (Figure 8a) points to the broadening of the polar part in both systems. However, this swelling is larger in [C_2_C_1_im][CF_3_SO_3_] than in [C_2_C_1_im][CH_3_SO_3_] and indicates a larger displacement of the fluorinated anion relative to the imidazolium cation due to the increasing number of interactions with water molecules. Figure 8b compares the structure factors for [C_2_C_1_im][C_4_F_9_SO_3_] and [C_2_C_1_im][C_4_H_9_SO_3_]. The PNPP at low-*q* values is the most conspicuous feature of the [C_2_C_1_im][C_4_F_9_SO_3_] systems and denotes a clear polar–nonpolar segregation in the system with fluorinated alkyl side chains [49]. Such PNPP is absent in [C_2_C_1_im][C_4_H_9_SO_3_] because the threshold for such segregation is higher in hydrogenated chains (C6 chains) than in the fluorinated ones (C4 chains) [49]. Moreover, the PNPP already seen in the pure fluorinated IL persists even at low concentrations of IL. Water swells the polar network of the IL (and eventually leads to its disruption), but the existence of segregated fluorinated domains persists even at higher water concentrations [51].

**Figure 9:**
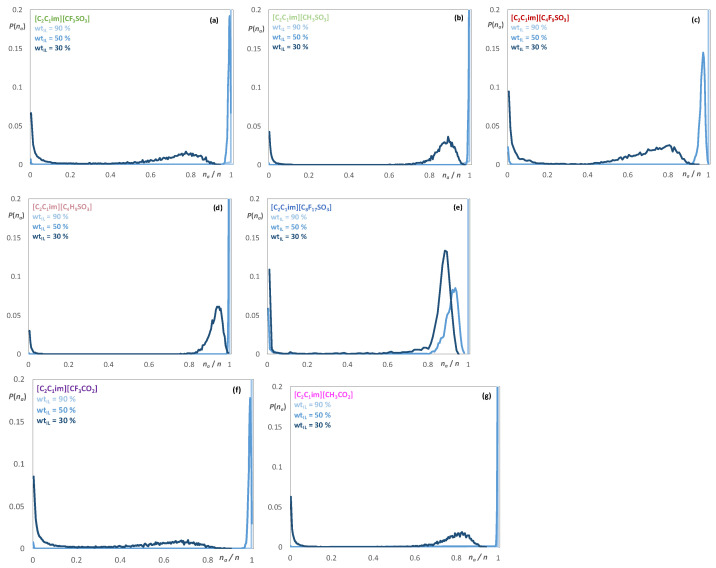
Discrete probability distribution of the cation–anion polar network for (**a**) [C_2_C_1_im][CF_3_SO_3_], (**b**) [C_2_C_1_im][CH_3_SO_3_], (**c**) [C_2_C_1_im][C_4_F_9_SO_3_], (**d**) [C_2_C_1_im][C_4_H_9_SO_3_], (**e**) [C_2_C_1_im][C_8_F_17_SO_3_], (**f**) [C_2_C_1_im][CF_3_CO_2_] and (**g**) [C_2_C_1_im][CH_3_CO_2_]. Data calculated at 300 K for aqueous solutions with wt_IL_ = 30% (X_IL_ = 0.01–0.04), wt_IL_ = 50% (X_IL_ = 0.05–0.10), and wt_IL_ = 90 % (X_IL_ = 0.23–0.49). The x-axes represent the ratio between the number of ionic species in an aggregate and the total number of those species in the simulation box. A (*n_a_*/*n*) value of 1 means that all ions in the simulation box belong to the same aggregate. Note that the pure and 90% wt_IL_ are represented by vertical lines at *n_a_*/*n* = 1.

In Figure 9, we show the extent of cation–anion aggregation within the polar networks of the ILs for the different concentrations of IL in aqueous solution (water–water aggregates are depicted in Figures S2–S5 of the Supplementary Materials). In all cases, there is a progressive disruption of the polar network as more water is present in the solutions. For the pure ILs and IL-rich solutions (wt_IL_ = 0.9; X_IL_ = 0.23–0.49), all ions are part of a single continuous network that permeates the entire simulation box: there is only one aggregate containing all ions of the simulation box (*P*(*n_a_*) = 1 for *n_a_*/*n* = 1). For wt_IL_ = 0.5 solutions (X_IL_ = 0.05–0.10), the probability of finding aggregates comprising all ions, *P*(*n_a_*), drops to 32% for [C_2_C_1_im][CH_3_SO_3_], 62% for [C_2_C_1_im][C_4_H_9_SO_3_], and 43% for [C_2_C_1_im][CH_3_CO_2_]). For the remaining ionic liquids, the *P*(*n_a_*) values are all under 10% and there is a shift towards finding very large aggregates that percolate the entire simulation box but that do not contain all ions present in the simulation (large *n_a_* values with *n_a_* < *n*). The most conspicuous case is the [C_2_C_1_im][C_8_F_17_SO_3_], where the distribution represents a definite shift toward larger aggregates that do not comprise all ions (although full percolation still exists most of the time, a few ion pairs detach from time to time from the (still) continuous polar network to form small satellite clusters). This is probably due to the presence of a large hydrophobic fluorinated side chain that pushes more water towards the polar network and disrupts cation–anion interactions more effectively. Finally, for wt_IL_ = 0.3 solutions (X_IL_ = 0.01–0.04), the *P*(*n_a_*) distributions continue to shift towards large aggregates that do not encompass all ion pairs with lower *n_a_* values. A closer look at the [C_2_C_1_im][C*_n_*F_2*n*+1_SO_3_] IL family (*n* = 1, 4 and 8) shows that, at these concentrations, ILs with longer fluorinated chains exhibit larger polar aggregates. In other words, for [C_2_C_1_im][C_8_F_17_SO_3_] disruption starts sooner than for [C_2_C_1_im][C_4_F_9_SO_3_] (cf. wt_IL_ = 0.5 lines in Figure 9c,e, please refer to the discussion in the previous paragraph), but it proceeds at a slower pace (cf. wt_IL_ = 0.3 lines in Figure 9c,e). Probably there is a rearrangement of the hydrophobic fluorinated chains to minimize the surface area exposed to water, resulting in large, fluorinated clusters (as indicated by the large PNPPs) that promote a partial preservation of their polar network. The comparison between [C_2_C_1_im][CF_3_SO_3_], [C_2_C_1_im][CH_3_SO_3_], [C_2_C_1_im][CF_3_CO_2_], and [C_2_C_1_im][CH_3_CO_2_] shows that the distributions for carboxylate ILs lead to slightly smaller polar network aggregates. This result is somehow expected since acetate is the stronger H-bond acceptor and forms more H-bonds than sulfonate [52]. Finally, if one compares systems with fluorinated chains with their hydrogenated counterparts, one sees a similar trend: the more hydrophobic fluorinated chains lead to smaller polar network aggregation numbers.

**Figure 10:**
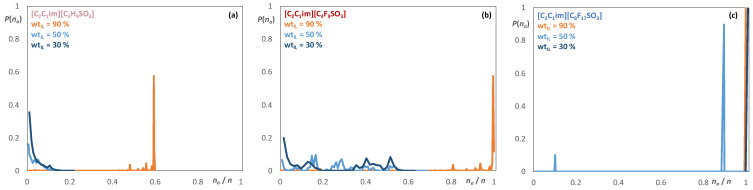
Discrete probability distribution of nonpolar aggregates for (**a**) [C_2_C_1_im][C_4_H_9_SO_3_], (**b**) [C_2_C_1_im][C_4_F_9_SO_3_], and (**c**) [C_2_C_1_im][C_8_F_17_SO_3_]. Data for aqueous solutions with wt_IL_ = 30 % (X_IL_ = 0.01–0.04), wt_IL_ = 50 % (X_IL_ = 0.05–0.10), and wt_IL_ = 90 % (X_IL_ = 0.23–0.49).

Figure 10 shows the nonpolar aggregation size distributions for the same IL systems (aqueous solutions with wt_IL_ = 0.9, 0.5, and 0.3). When comparing hydrogenated IL systems with their fluorinated counterparts (Figure 10a,b), it is evident that the nonpolar aggregates are larger in the fluorinated ILs. Moreover, the aggregate analysis points to a distribution of aggregates for [C_2_C_1_im][C_4_F_9_SO_3_] for wt_IL_ = 0.3 (X_IL_ = 0.02, Figure 10b) that still does not percolate the simulation box, with the largest aggregate containing 63% of the total chains. In contrast, the aggregation behavior of [C_2_C_1_im][C_8_F_17_SO_3_], with longer fluoroalkyl chains, indicates that the critical aggregation concentration was already reached before wt_IL_ = 0.3 (X_IL_ = 0.01, Figure 10c). Here, the formation of a single aggregate is the result of induced-dipole/induced-dipole dispersion interactions between fluoroalkyl chains and the hydrophobic effect, rather than percolation through the simulation box.

**Figure 11:**
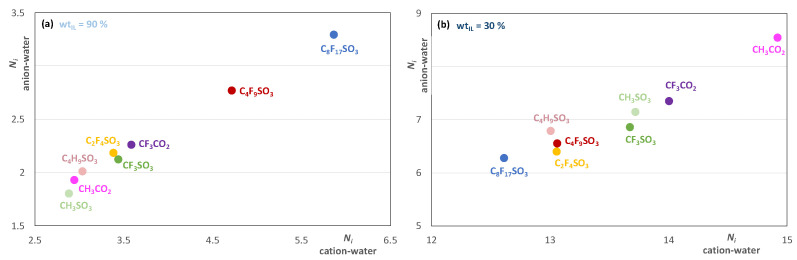
Average number of water neighbors around the cation vs. the anion for the [C_2_C_1_im][C*_n_*F_2*n*+1_SO_3_], [C_2_C_1_im][C*_n_*H_2*n*+1_SO_3_], [C_2_C_1_im][CF_3_CO_2_], and [C_2_C_1_im][CH_3_CO_2_] at (**a**) wt_IL_ = 90 (X_IL_ = 0.23–0.49) and (**b**) wt_IL_ = 30 % (X_IL_ = 0.01–0.04).

The average water neighbors around the cation vs. the anion for the [C_2_C_1_im][C*_n_*F_2*n*+1_SO_3_], [C_2_C_1_im][C*_n_*H_2*n*+1_SO_3_], [C_2_C_1_im][CF_3_CO_2_], and [C_2_C_1_im][CH_3_CO_2_] ILs at wt_IL_= 0.9 and 0.3 are depicted in Figure 11. In all cases, more water molecules surround the cation than the charged moiety of the anion. This can be explained by the different size and degree of charge delocalization of each type of ion. Moreover, there is a strong correlation between the two quantities, implying that water molecules move into positions adjacent to the polar network and can interact with both ions. At higher water concentrations, the number of water molecules per ion is obviously higher. The data shows that carboxylate anions are more solvated than their sulphonate counterparts (cf. [C_2_C_1_im][CF_3_SO_3_] versus [C_2_C_1_im][CF_3_CO_2_] and [C_2_C_1_im][CH_3_SO_3_] versus [C_2_C_1_im][CH_3_CO_2_] in both Figure 11a,b). This is due to the more hydrophilic character of the carboxylate moiety [53]: the oxygen atoms of carboxylate are modeled using partial charges of −0.80 a.c.u., whereas those of sulfonate are modeled using partial charges of −0.63 a.c.u.

The length and nature (hydrogenated versus fluorinated) of the alkyl side chains play a similar role in the water solvation process: at modest water concentrations (wt_IL_ = 0.9; X_IL_ = 0.23–0.49), the existence of longer and more fluorinated chains leads to more interactions between water molecules and the polar network (as we have discussed before, [C_2_C_1_im][C_8_F_17_SO_3_] is the first to lose the integrity and continuity of its polar network and start the formation of large polar network clusters); at higher water concentrations (wt_IL_= 0.3; X_IL_ = 0.01–0.04), the existence of nonpolar aggregates (more pronounced in longer and fluorinated chains) leads to relatively low numbers of water–ion interactions (disruption in [C_2_C_1_im][C_8_F_17_SO_3_] starts earlier but proceeds at a slower pace).

**Figure 12:**
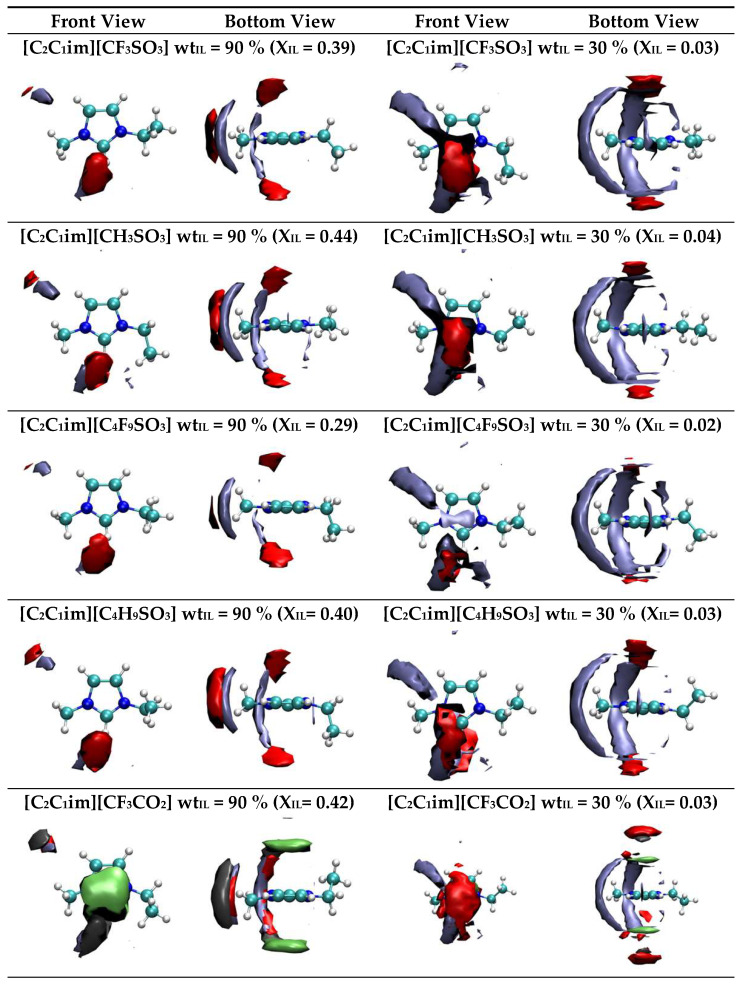

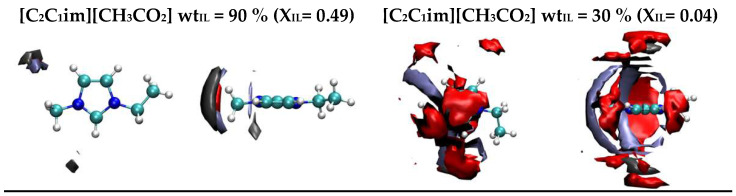
Selected spatial distribution functions around the [C_2_C_1_im]^+^ cation in aqueous mixtures of [C_2_C_1_im][C*_n_*F_2*n*+1_SO_3_], [C_2_C_1_im][C*_n_*H_2*n*+1_SO_3_], [C_2_C_1_im][CF_3_CO_2_], and [C_2_C_1_im][CH_3_CO_2_] at 300 K. The grey, red, and green colors represent C, O (-CO_2_)/S (-SO_3_) and F atoms of anion, respectively, and light blue depicts water O atom. The isosurface value is 70% of the maximum number density.

The spatial distribution functions, SDFs, for selected atoms around the [C_2_C_1_im]^+^ cation for IL aqueous solutions with wt_IL_ = 0.9 and 0.3 in the [C_2_C_1_im][C*_n_*F_2*n*+1_SO_3_], [C_2_C_1_im][C*_n_*H_2*n*+1_SO_3_], [C_2_C_1_im][CF_3_CO_2_], and [C_2_C_1_im][CH_3_CO_2_] systems are presented in Figure 12. Results for wt_IL_ = 0.5 (X_IL_ = 0.05–0.10) are presented in Figures S6–S9 of the Supplementary Materials. The patterns of interaction of the sulfonate ions and water molecules around the imidazolium cations are strikingly similar for each concentration. It must be stressed that the isodensity surface plots do not show how high the density is inside each contour; they only provide a spatial overview of the locations of the most probable interactions. At wt_IL_ = 0.9 (X_IL_ = 0.23–0.49), both the fluorinated and hydrogenated R-SO_3_^−^ anions are in clear competition with water molecules for the acidic hydrogens at the C2 and C4 positions of [C_2_C_1_im]^+^. The anions are gradually displaced from these preferred coordination sites as the amount of water increases, in line with the observed shifts of the COPs to lower *q* values in the *S*(*q*) functions. At wt_IL_ = 0.3 (X_IL_ = 0.01–0.04), the SDFs show that water molecules can occupy a ring region around the methyl group of the cation, spanning both acidic hydrogens at C2 and C4. Interestingly, there is less competition for the C5 H atom, probably due to partial steric hindrance caused by the ethyl chain of the cation.

Regarding the carbonate-based anions, the SDFs show some differences in spatial organization around the cations relative to the sulfonate-based ions, namely at different compositions. Similar to their sulfonate-based counterparts, fluorinated and hydrogenated R-CO_2_^−^ anions are also in competition with water for the hydrogen atoms at the C2 and C4 positions of the cation; however, the anion–cation SDFs change dramatically upon addition of water. At wt_IL_ = 0.9 (X_IL_ = 0.49), the C-C axis of the [CF_3_CO_2_]^−^ ion is parallelly oriented with the plane of the imidazolium ring and the cations are intercalated by the CF_3_ group of anions. With the increase in water concentration to wt_IL_ = 0.3 (X_IL_ = 0.03), the [CF_3_CO_2_]^−^ ions are displaced from the vicinity of the acidic hydrogens and adopt a perpendicular position with respect to the plane of the imidazolium cation, with the CF_3_ group pointing towards the ring. These findings are corroborated by the depiction of additional cation–anion SDFs corresponding to distribution functions between the cation center-of-charge and the carbon and fluorine atoms of the anion (grey and green contours in Figure 12, respectively). On the other hand, water molecules form strong H-bonds with the [CH_3_CO_2_]^−^ ion, displacing it not only from the vicinity of the acidic hydrogens of the cation but also from the cation’s coordination sphere. At wt_IL_ = 0.3 (X_IL_ = 0.04), SDFs indicate that the [CH_3_CO_2_]^−^ ion is involved in their own solvation sphere (as pointed out by average water neighbors in Figure 11b) and can be found anywhere around the polar moiety of the cation. The CH_2_ group in the alkyl chain is not affected by the addition of water, in agreement with previous NMR results [54].

**Figure 13:**
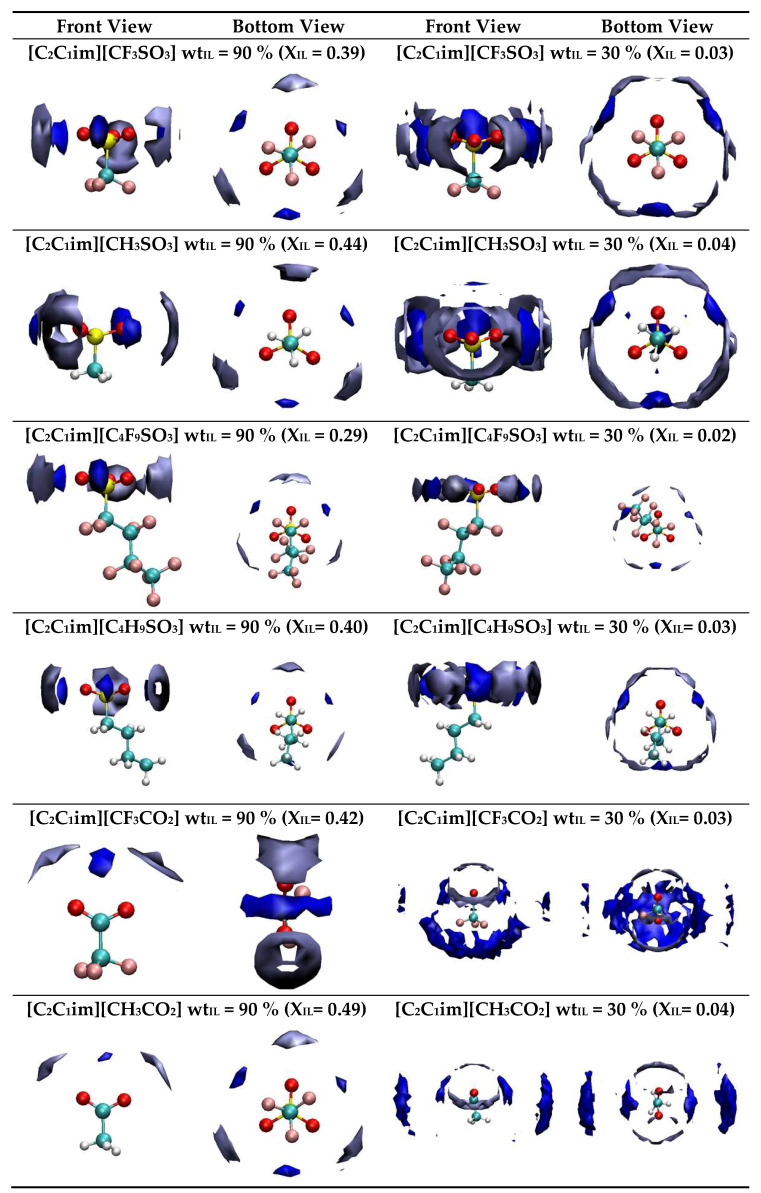
Selected spatial distribution functions around the [C*_n_*F_2*n*+1_SO_3_]^−^, [C*_n_*H_2*n*+1_SO_3_]^−^, [CF_3_CO_2_]^−^, and [CH_3_CO_2_]^−^ anions in aqueous mixtures of [C_2_C_1_im][C*_n_*F_2*n*+1_SO_3_], [C_2_C_1_im][C*_n_*H_2*n*+1_SO_3_], [C_2_C_1_im][CF_3_CO_2_], and [C_2_C_1_im][CH_3_CO_2_] at 300 K. The blue color represents H atom of cations attached to the carbon between nitrogen atoms of the imidazolium ring and light blue depicts water O atom. The isosurface value is 70% of the maximum number density.

The SDFs for the H atom at C2 of the cation and the oxygen atom of water around different anions for IL aqueous solutions with wt_IL_ = 0.9 and 0.3 in the [C_2_C_1_im][C*_n_*F_2*n*+1_SO_3_], [C_2_C_1_im][C*_n_*H_2*n*+1_SO_3_], [C_2_C_1_im][CF_3_CO_2_], and [C_2_C_1_im][CH_3_CO_2_] systems are presented in Figure 13. Results for wt_IL_ = 0.5 (X_IL_ = 0.05–0.10) are presented in Figures S10–S13 in Supplementary Materials. It is very clear that water molecules and the most acidic proton of the [C_2_C_1_im]^+^ ion compete heavily for the regions around the oxygen atoms of the anions. The three-fold and two-fold symmetries of the sulfonate and carboxylate headgroups are also quite well captured in the SDFs. Obviously, the increase in water content produces SDFs with water present in larger areas around the anions. However, the SDFs also show that the cations are never fully displaced, corroborating the persistence of the COPs in the simulated *S*(*q*) functions. For the carboxylate anions, the behavior is also similar when the water content is low. However, for wt_IL_ = 0.3 (X_IL_ = 0.04), the [CH_3_CO_2_]^−^ ion is fully surrounded by water, whereas the [CF_3_CO_2_]^−^ ion is oriented with its CF_3_ group pointing to the cation and its carboxylate moiety being surrounded by water.

### 2.3. Aqueous Solutions of [N_1 2OH 2OH 2OH_][NTf_2_]

The last system to be considered is an ionic liquid that combines a cation that can interact strongly with water, [N_1 2OH 2OH 2OH_]^+^, with a hydrophobic anion, [NTf_2_]^–^. In this case, the ionic liquid is completely miscible with water, and we have conducted simulations at concentrations ranging from the pure IL to pure water.

**Figure 14:**
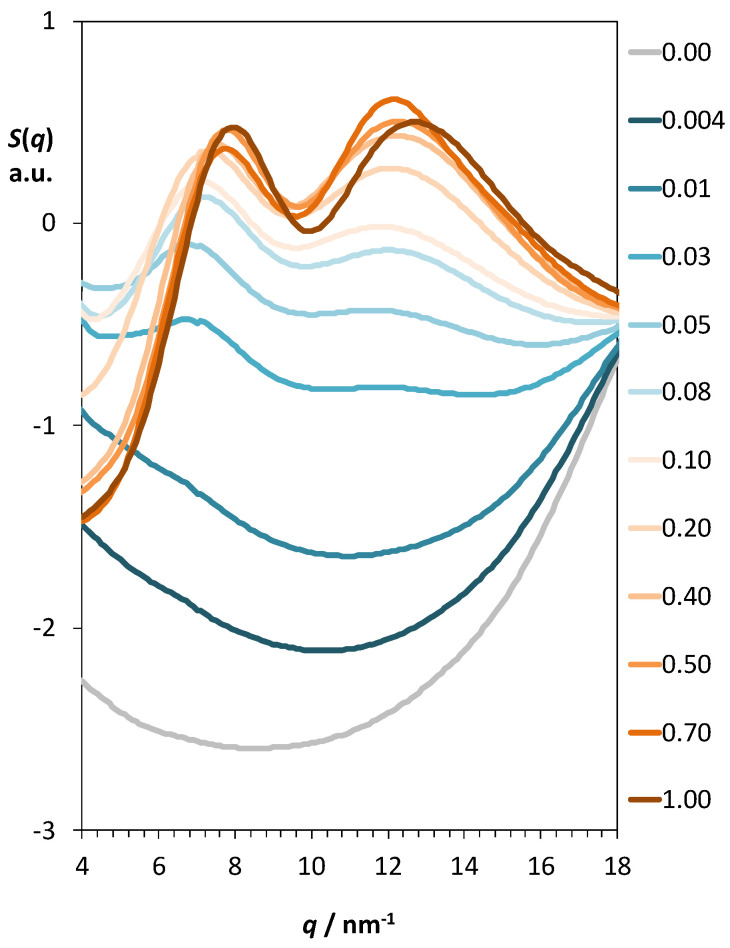
Total structure factor functions, *S*(*q*) in arbitrary units, of [N_1 2OH 2OH 2OH_][NTf_2_] aqueous mixtures as a function of mole fraction of water.

**Figure 15:**
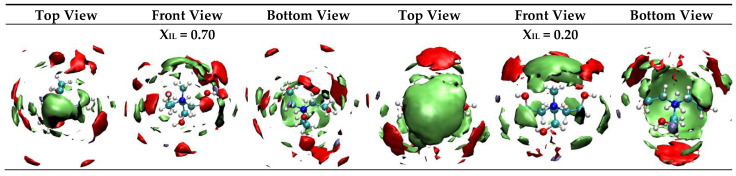

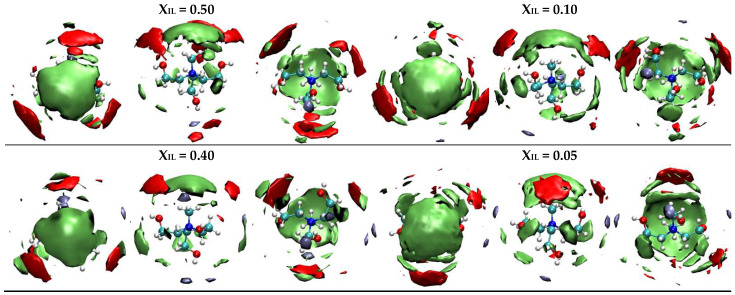
Selected spatial distribution functions around the [N_1 2OH 2OH 2OH_]^+^ cation in aqueous mixtures of [N_1 2OH 2OH 2OH_][NTf_2_] at 300 K. The red and green colors represent N and F atoms of anion, respectively, and light blue depicts the water O atom. The isosurface value is 70% of the maximum number density.

Figure 14 shows calculated structure factor functions of aqueous solutions of [N_1 2OH 2OH 2OH_][NTf_2_] and Figure 15 depicts selected SDFs around the nitrogen atom of the cation. At X_IL_ = 0.70, the SDFs show that the anions are distributed around the cation, and the structure factor function indicates a small increase in characteristic distances within the polar network (the COP *q* value decreases) in comparison to the pure IL. Moreover, an incipient preferential orientation of the fluorine atoms of the anion towards the cation methyl group can be noticed. At this concentration, an analysis of the size of water aggregates (Figure S14 in Supplementary Materials) indicated that water molecules are present in solution mostly as monomers (60%) or dimers (27%). The characteristic nanostructure organization of [N_1 2OH 2OH 2OH_][NTf_2_], with cations and anions forming a continuous network of charge-alternating ions, are not disrupted by the presence of water molecules until X_IL_ = 0.03 [28] (the COP is still obvious at that concentration, Figure 14).

Ludwig et al. [55,56] have extensively reported on the cooperative behavior of hydrogen bonding in ionic liquids. The conventional rules of charge attraction/repulsion are not completely observed in ILs, leading to the formation of H-bonded cationic clusters that are governed by the charge delocalization of the cation. As the mole fraction of water increases, the original cation OH-OH hydrogen bond network is replaced by water–cation H-bond interactions [28,30]. The [NTf_2_]^−^ anions are gradually displaced to the vicinity of the CH_3_ group of the cation, but they remain in electrostatic close contact since the charge in cholinium cations is mainly localized on the N atom and its four adjoining CH_3_/CH_2_ groups. This rearrangement of the anions around the cations also supports the small decrease in *q* values of the COPs in the calculated structure factor functions presented in Figure 14.

**Figure 16:**
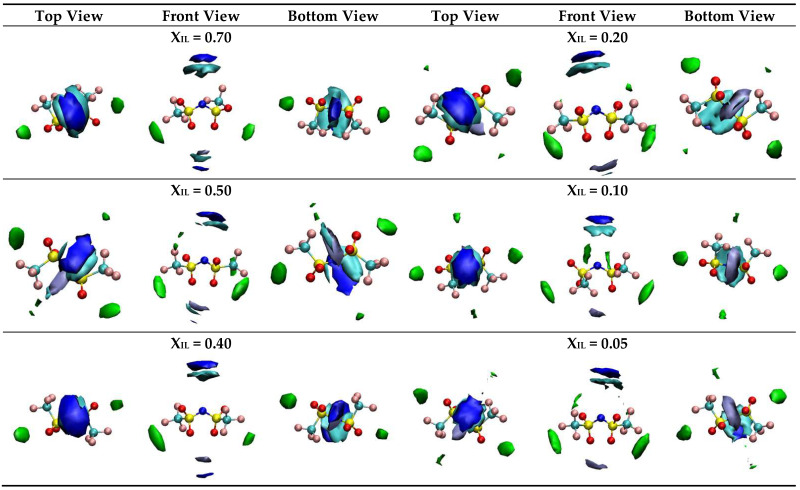
Selected spatial distribution functions around the [NTf_2_]^−^ anion in aqueous mixtures of [N_1 2OH 2OH 2OH_][NTf_2_] at 300 K. The blue and cyan colors represent cation N atom and CH_3_ group, respectively, green denotes F atoms of anion and light blue depicts water O atom. The isosurface value is 70% of the maximum number density.

The SDFs between the nitrogen atom of the anion and selected atoms of the cation and the oxygen atom of water are presented in Figure 16. The cations are distributed above and below the S-N-S plane of the anion. Other anions interact via their –CF_3_ groups along the plane. The simulation results also indicate that the water molecules interact with the anion mainly through the oxygen atoms of the sulfonate groups that are in axial positions relative to the S-N-S plane (for both possible conformers of the anion). As X_IL_ decreases, the cations are gradually displaced to the vicinity equatorial region of the S-N-S plane [28].

**Figure 17:**
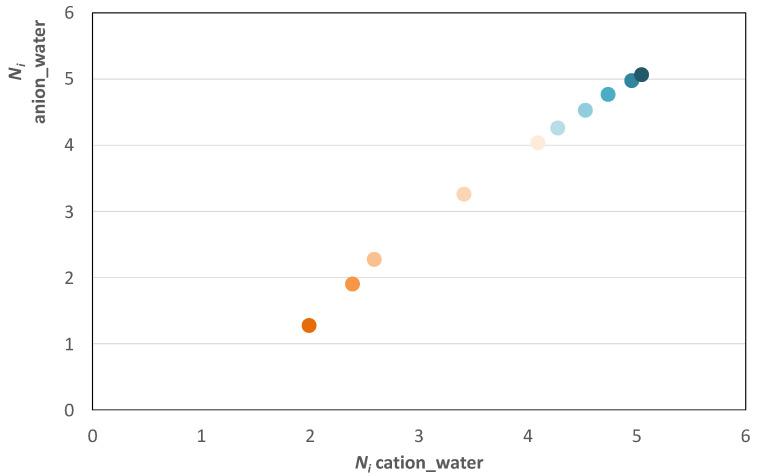
Average number of neighbors around the cation (*N_i_* cation_water) vs. the anion (*N_i_* anion_water) in aqueous solutions of [N_1 2OH 2OH 2OH_][NTf_2_].

The average number of contact water molecules for the [N_1 2OH 2OH 2OH_] and [NTf_2_] ions is shown in Figure 17. As expected, the values are slightly larger for the cation. This result, combined with the subtle changes in SDFs and structure factors, indicate that the hydrophobic nature of the anion combined with the electrostatic interactions with the cation are strong enough to keep both ions within the same solvation cage.

## 3. Computational Details

Molecular dynamics (MD) simulations of the pure ionic liquids and their aqueous mixtures were carried out using the DLPOLY and GROMACS packages [57,58,59,60,61,62]. Initial configurations were built with Packmol software [63]. Water molecules and all Ils were modelled using, respectively, the SPC model [64] and the previously described CL&P all atom force field [11,12], which is based on the OPLS-AA framework [65] and has been thoroughly developed specifically to encompass several IL families. The MD simulations for each ionic liquid and respective aqueous mixtures started from low-density initial configurations.

The number of ion pairs and box length for all Ils and their aqueous mixtures are presented in Table 1, as well as the weight and mole fractions of the Ils. The IL volume fraction closely follows the mass fraction; therefore, we opted to not add this data. It is important to stress that all the IL–water compositions selected for this study are homogeneous mixtures at 300 or 320 K. The boxes were equilibrated under isothermal–isobaric ensemble conditions for 1 ns at 300 or 320 K and 1 atm using the Nosé–Hoover thermostat and isotropic barostat with time constants of 0.5 and 2 ps, respectively. Simulation runs of at least 4 ns were used to produce equilibrated systems at the studied temperature. Electrostatic interactions were treated using the Ewald summation method considering six reciprocal-space vectors, and repulsive–dispersive interactions were explicitly calculated below a cut-off distance of 1.6 nm (long-range corrections were applied assuming the system has a uniform density beyond that cut-off radius). Further information can be found elsewhere [66].

## 4. Conclusions

**Figure 18:**
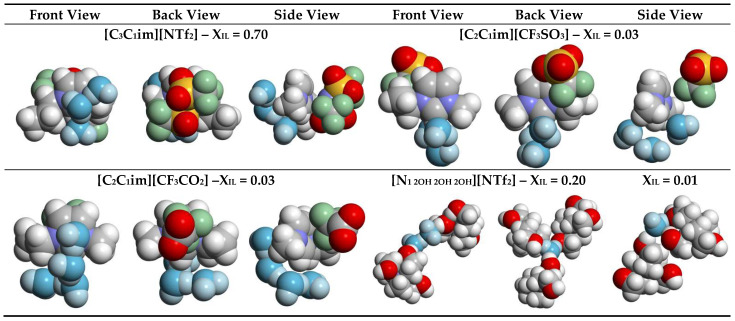
Examples of aggregates and clusters formed by cations, anions, and water molecules cooperating via hydrogen bonding and F-π interactions, found in MD simulation trajectories of the several studied systems. Atoms of cations and anions are depicted in CPK colors and water atoms are represented in blue hues.

In Figure 18, representative examples of the most significative aggregates found in some of the studied systems are shown. In systems with imidazolium cations and small fluorinated anions, such as [C_3_C_1_im][NTf_2_], [C_4_C_1_im][NTf_2_], [iC_4_C_1_im][NTf_2_], [C_2_C_1_im][CF_3_SO_3_], and [C_2_C_1_im][CF_3_CO_2_], the F-π interactions between the anion and the imidazolium ring hold both cation and anion within the same solvation sphere. The most unusual behavior was observed for the strong hydrogen-bond acceptor [CF_3_CO_2_]^−^ anion: at high water concentration, the anion is displaced from the vicinity of the acidic hydrogens and adopts a perpendicular position with respect to the plane of the imidazolium cation, with the CF_3_ group pointing towards the ring. Moreover, the aggregate distributions between water and polar moieties in these systems with low water content indicated that water molecules interact strongly with the cations. With respect to the formation of nonpolar aggregates, the most interesting behavior was observed for the perfluoroalkyl chains of the [C_2_C_1_im][C_8_F_17_SO_3_] ionic liquid, which forms a single aggregate at X_IL_ = 0.01.

The hydrophilicity of the [N_1 2OH 2OH 2OH_]^+^ can promote the solubilization of highly hydrophobic anions such as [NTf_2_]^−^ in all proportions with water. The strong cation–anion coulombic attraction combined with the hydrophobic effect of the anion holds the two ions within the same solvation shell. Moreover, water can intermediate the cluster formation by bridging two or three cations or connecting two cations where two OH groups of the same cation contribute to the cluster formation.

The water–water aggregates shown in Figures S1–S5 and S14 (Supplementary Material) portray an interesting trend. In the IL-rich mixtures, i.e., at low water concentrations (X_IL_ > 0.5), the water molecules are dispersed as monomers or forming small, chainlike aggregates H-bonded to the polar moieties of the ionic liquids. As the water amount increases, the monomeric water and its small aggregates coalesce into a continuous phase. On the other hand, the nature of the ionic liquid subphase (or pseudophase) will be dictated by its hydrogen bonding ability. The coulombic network of hydrophilic ILs with strong coordinating anions such as Cl^−^ or [CH_3_CO_2_]^−^ can be disrupted by water [48], while hydrophobic ILs develop biphasic systems.

Unfortunately, synthetic chemists depend greatly on organic solvents because many substances involved in chemical reactions are insoluble in water. Therefore, agents that act as solubilizers are needed to achieve a successful transfer of such chemical reactions to aqueous media. One can envisage the application of these functionalized ILs as additives that increase the solubility of reactants in water, capable of forming aggregates with different hydrophobic domains, in a similar mechanism performed by micelles [67,68,69].

## Figures and Tables

**Table 1:** Details of the molecular dynamics simulations performed for the water–ionic liquid mixtures and the corresponding sections where the systems are discussed. The final box length at 300 or 320 K is represented by *l_box_* (in nm); the number of IL pairs and water molecules are, respectively, *n_IL_* and *n_water_*; the IL weight fraction is represented by wt_IL_; and the IL mole fraction is denoted as X_IL_.

Section	Ionic Liquid	*n_IL_*	*n_water_*	wt_IL_	X_IL_	*l_box_*	Section	Ionic Liquid	*n_IL_*	*n_water_*	wt_IL_	X_IL_	*l_box_*
Section 2.1	[C_3_C_1_im][NTf_2_]	320	–	1.00	1.00	5.19	Section 2.2	[C_2_C_1_im][C_4_H_9_SO_3_]	600	–	1.00	1.00	6.07
	[C_4_C_1_im][NTf_2_]	300	–	1.00	1.00	5.28		[C_2_C_1_im][C_4_H_9_SO_3_]	420	620	0.90	0.40	5.57
	[iC_4_C_1_im][NTf_2_]	300	–	1.00	1.00	5.20		[C_2_C_1_im][C_4_H_9_SO_3_]	190	2600	0.50	0.07	5.26
	[C_3_C_1_im][NTf_2_]	300	130	0.98	0.70	5.16		[C_2_C_1_im][C_4_H_9_SO_3_]	105	3300	0.30	0.03	5.14
	[C_4_C_1_im][NTf_2_]	300	130	0.98	0.70	5.25		[C_2_C_1_im][CF_3_CO_2_]	1000	–	1.00	1.00	6.55
	[iC_4_C_1_im][NTf_2_]	300	130	0.98	0.70	5.25		[C_2_C_1_im][CF_3_CO_2_]	450	620	0.90	0.42	5.27
Section 2.2	[C_2_C_1_im][CF_3_SO_3_]	1000	–	1.00	1.00	6.69		[C_2_C_1_im][CF_3_CO_2_]	210	2600	0.50	0.07	5.13
	[C_2_C_1_im][CF_3_SO_3_]	390	620	0.90	0.39	5.16		[C_2_C_1_im][CF_3_CO_2_]	115	3300	0.30	0.03	5.05
	[C_2_C_1_im][CF_3_SO_3_]	180	2600	0.50	0.06	5.08		[C_2_C_1_im][CH_3_CO_2_]	1000	–	1.00	1.00	6.39
	[C_2_C_1_im][CF_3_SO_3_]	100	3300	0.30	0.03	5.57		[C_2_C_1_im][CH_3_CO_2_]	600	620	0.90	0.49	5.56
	[C_2_C_1_im][C_4_F_9_SO_3_]	350	–	1.00	1.00	5.30		[C_2_C_1_im][CH_3_CO_2_]	280	2600	0.50	0.10	5.25
	[C_2_C_1_im][C_4_F_9_SO_3_]	250	620	0.90	0.29	5.00		[C_2_C_1_im][CH_3_CO_2_]	150	3300	0.30	0.04	5.11
	[C_2_C_1_im][C_4_F_9_SO_3_]	115	2600	0.50	0.05	5.01	Section 2.3	[N_1 2OH 2OH 2OH_][NTf_2_]	350	–	1.00	1.00	5.44
	[C_2_C_1_im][C_4_F_9_SO_3_]	62	3300	0.30	0.02	4.98		[N_1 2OH 2OH 2OH_][NTf_2_]	340	146	0.98	0.70	5.40
	[C_2_C_1_im][C_8_F_17_SO_3_]	250	–	1.00	1.00	5.32		[N_1 2OH 2OH 2OH_][NTf_2_]	320	320	0.96	0.50	5.40
	[C_2_C_1_im][C_8_F_17_SO_3_]	180	620	0.90	0.23	5.00		[N_1 2OH 2OH 2OH_][NTf_2_]	320	480	0.94	0.40	5.44
	[C_2_C_1_im][C_8_F_17_SO_3_]	78	2600	0.50	0.05	4.97		[N_1 2OH 2OH 2OH_][NTf_2_]	280	1120	0.86	0.20	5.46
	[C_2_C_1_im][C_8_F_17_SO_3_]	43	3300	0.30	0.01	4.97		[N_1 2OH 2OH 2OH_][NTf_2_]	220	1980	0.73	0.10	5.44
	[C_2_C_1_im][CH_3_SO_3_]	1000	–	1.00	1.00	6.62		[N_1 2OH 2OH 2OH_][NTf_2_]	160	3040	0.56	0.05	5.50
	[C_2_C_1_im][CH_3_SO_3_]	490	620	0.90	0.44	5.42		[N_1 2OH 2OH 2OH_][NTf_2_]	115	3718	0.43	0.03	5.51
	[C_2_C_1_im][CH_3_SO_3_]	230	2600	0.50	0.08	5.22		[N_1 2OH 2OH 2OH_][NTf_2_]	48	4752	0.20	0.01	5.45
	[C_2_C_1_im][CH_3_SO_3_]	125	3300	0.30	0.04	5.11		[N_1 2OH 2OH 2OH_][NTf_2_]	20	4980	0.09	0.004	5.20

## Data Availability

Not applicable.

## Supplementary Materials

The following are available online, Figures S1–S5 and S14: Discrete distribution of aggregate sizes of aqueous solutions of studied systems, Figures S6–S13: Selected spatial distribution functions.

## Sample Availability

Force field parameters and output data files are available from the authors.
