# Supplementary material for: Ionic Liquids and Water: Hydrophobicity vs. Hydrophilicity
Source: Molecules. 2021 Nov 26;26(23):7159. doi: 10.3390/molecules26237159 (PMC8658901; doi:10.3390/molecules26237159)
Supplement: Supplementary file 1 [file molecules-26-07159-s001.zip › molecules-1458316-supplementary.pdf]

Supplementary Materials
for

Ionic Liquids and water: hydrophobicity *vs* hydrophilicity

Rita F. Rodrigues [1], Adilson A. Freitas [1,*], José N. Canongia Lopes [1] and Karina Shimizu [1,*]

Centro de Química Estrutural, Instituto Superior Técnico, Universidade de Lisboa, 1049-001 Lisbon, Portugal

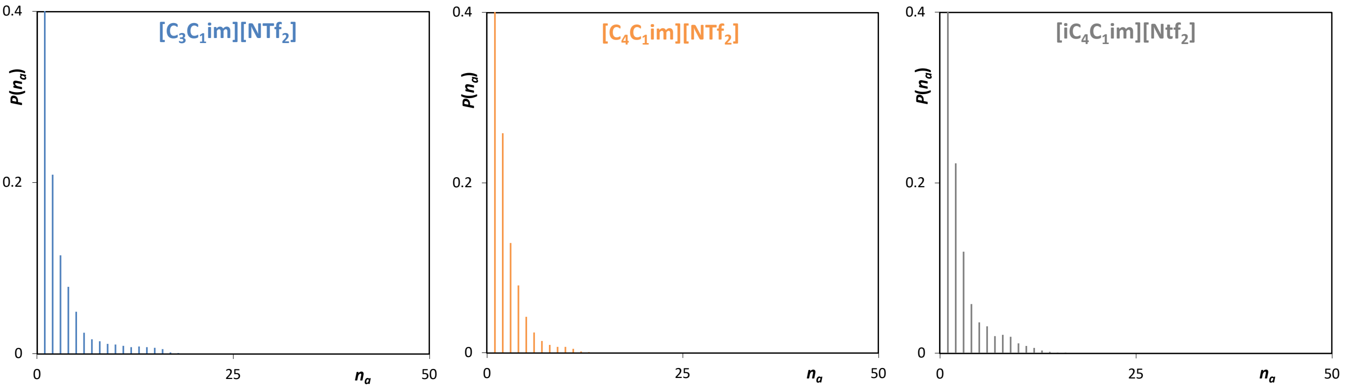

Figure S1. Discrete probability distribution of aggregate sizes, $P(n_a)$, as a function of aggregate size number, n_a, for water-water aggregates found in aqueous solutions of $[C_3C_1im][NTf_2]$, $[C_4C_1im][NTf_2]$ and $[iC_4C_1im][NTf_2]$ with $X_{IL} = 0.70$ at 320 K.

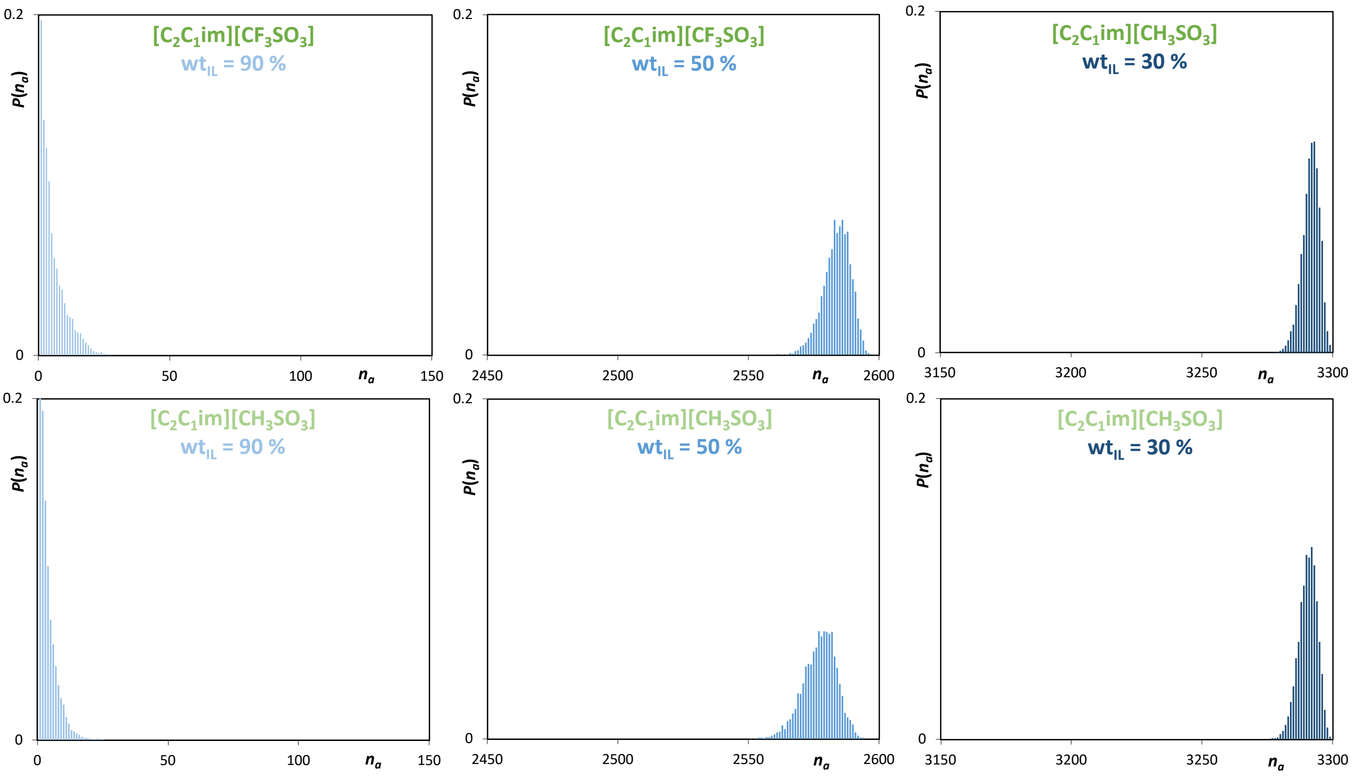

Figure S2. Discrete probability distribution of aggregate sizes, $P(n_a)$, as a function of aggregate size number, n_a, for water-water aggregates found in aqueous solutions of [C_2C_1im][CF_3SO_3] and [C_2C_1im][CH_3SO_3] at 300 K.

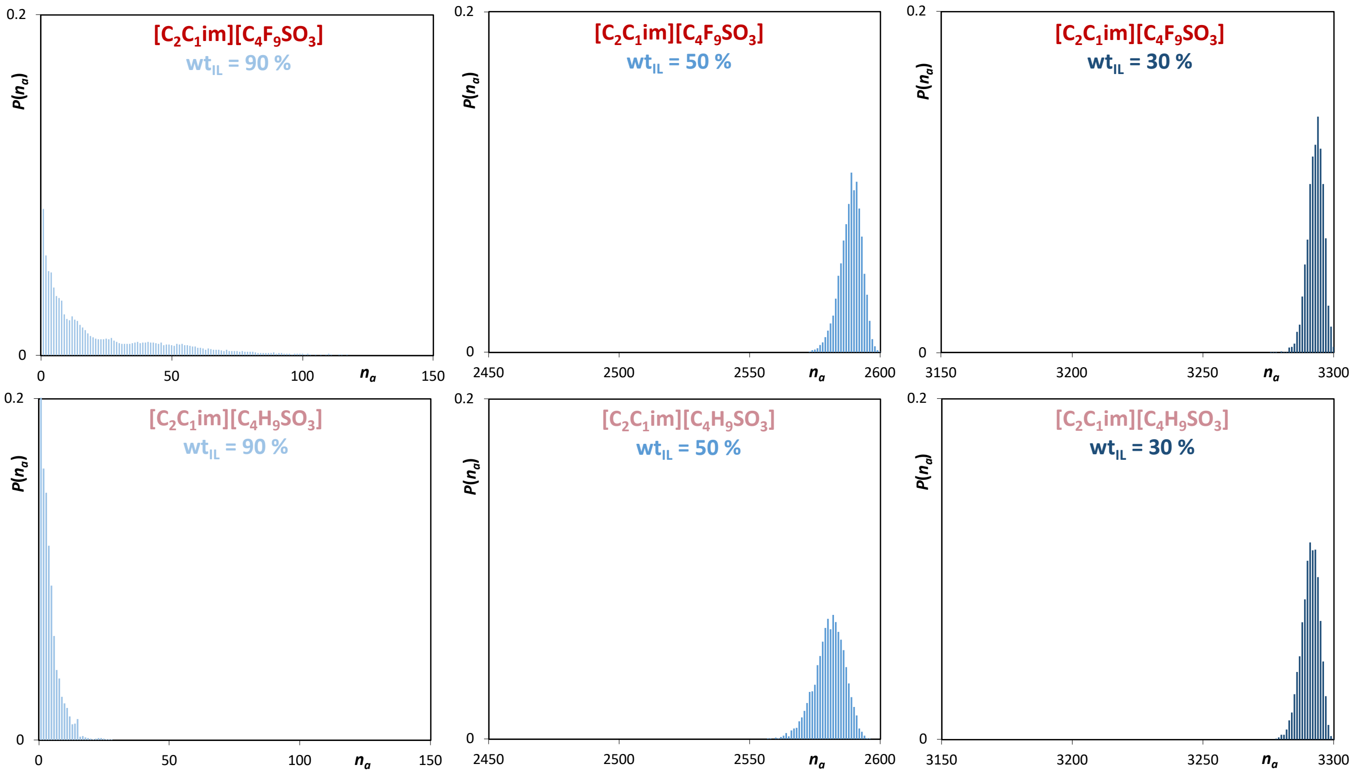

Figure S3. Discrete probability distribution of aggregate sizes, $P(n_a)$, as a function of aggregate size number, n_a, for water-water aggregates found in aqueous solutions of $[C_2C_1im][C_4F_9SO_3]$ and $[C_2C_1im][C_4H_9SO_3]$ at 300 K.

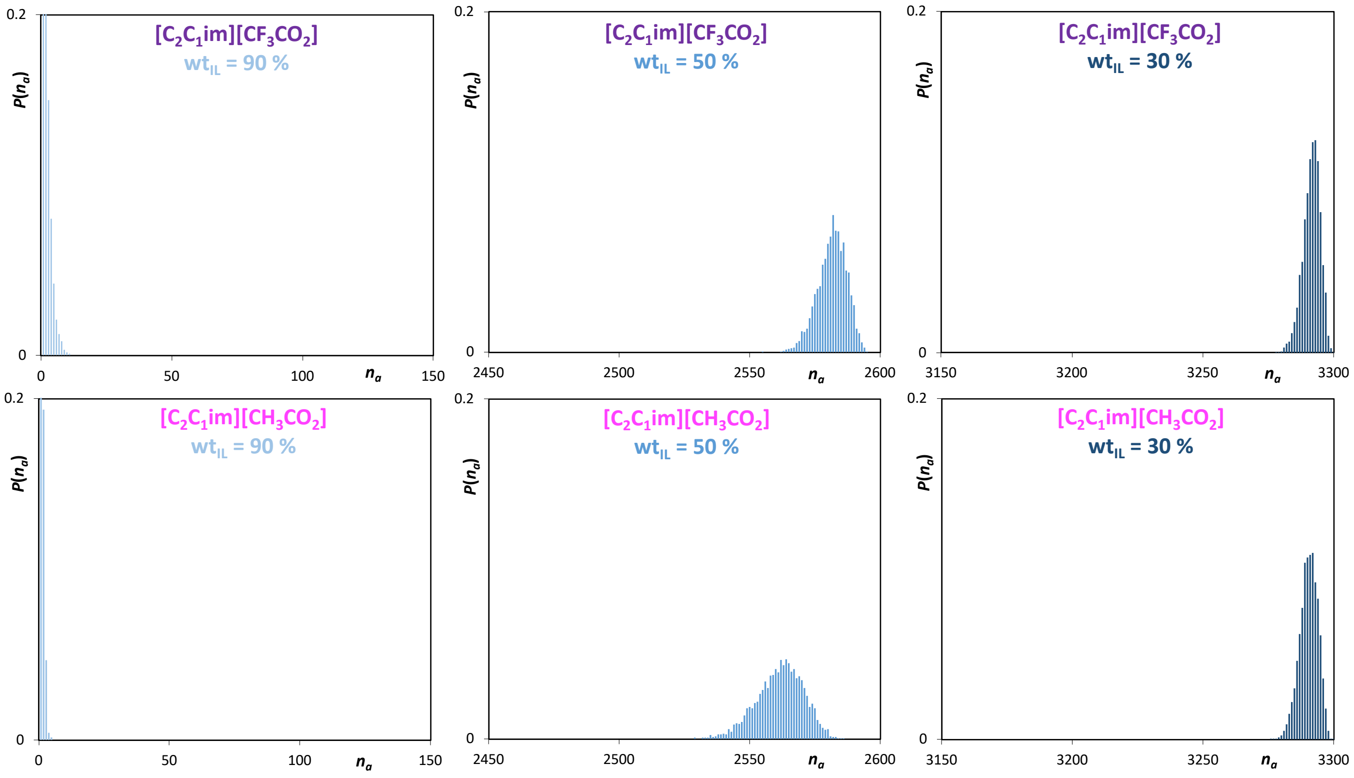

Figure S4. Discrete probability distribution of aggregate sizes, $P(n_a)$, as a function of aggregate size number, n_a, for water-water aggregates found in aqueous solutions of $[C_2C_1im][CF_3CO_2]$ and $[C_2C_1im][CH_3CO_2]$ at 300 K.

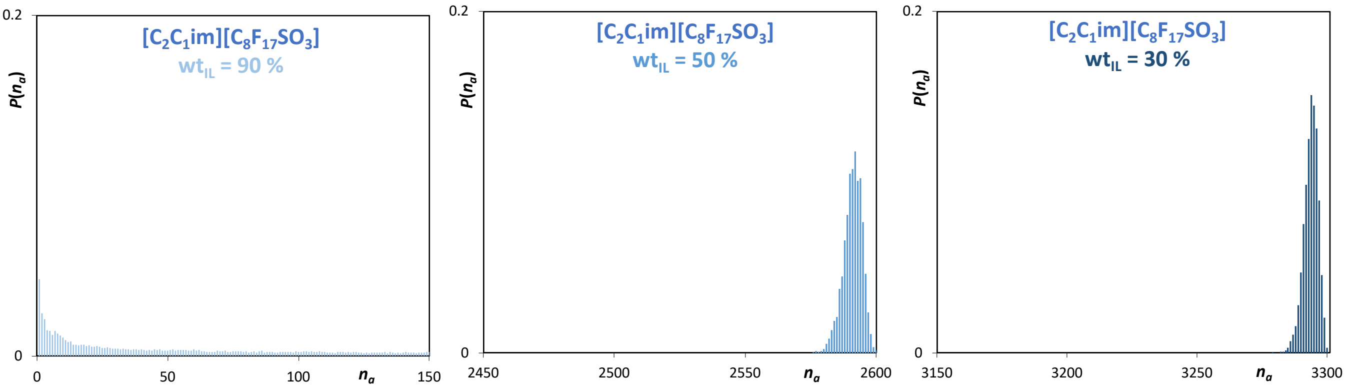

Figure S5. Discrete probability distribution of aggregate sizes, $P(n_a)$, as a function of aggregate size number, n_a, for water-water aggregates found in aqueous solutions of [C_2C_1im][$C_8F_{17}SO_3$] at 300 K.

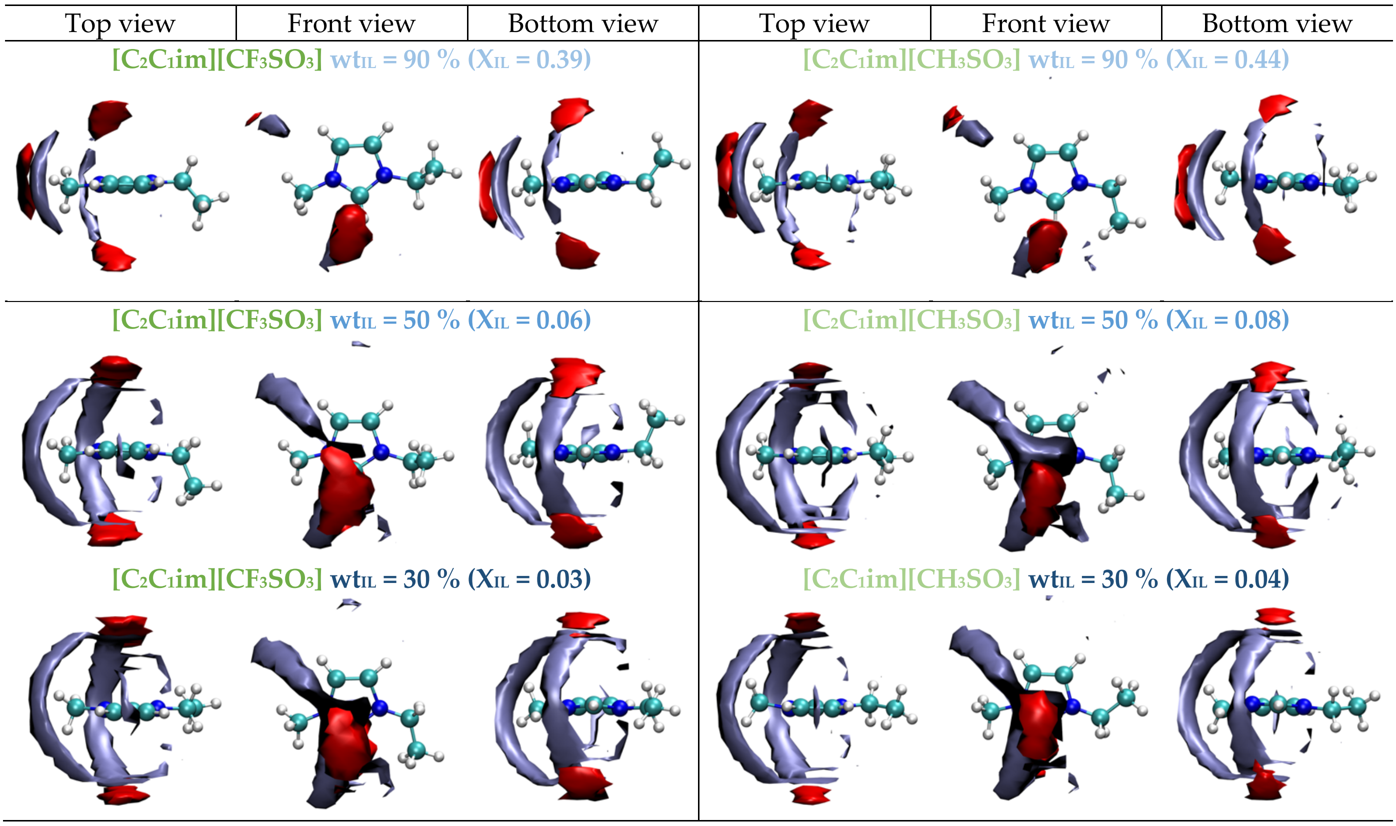

Figure S6. Selected Spatial Distribution Functions around the [C_2C_1im]$^+$ cation in aqueous mixtures of [C_2C_1im][CF_3SO_3] and [C_2C_1im][CH_3SO_3] at 300 K. The red and light blue colours represent S (-<u>S</u>O_3) atom of anion and water O atom, respectively. The isosurface value is 70% of the maximum number density.

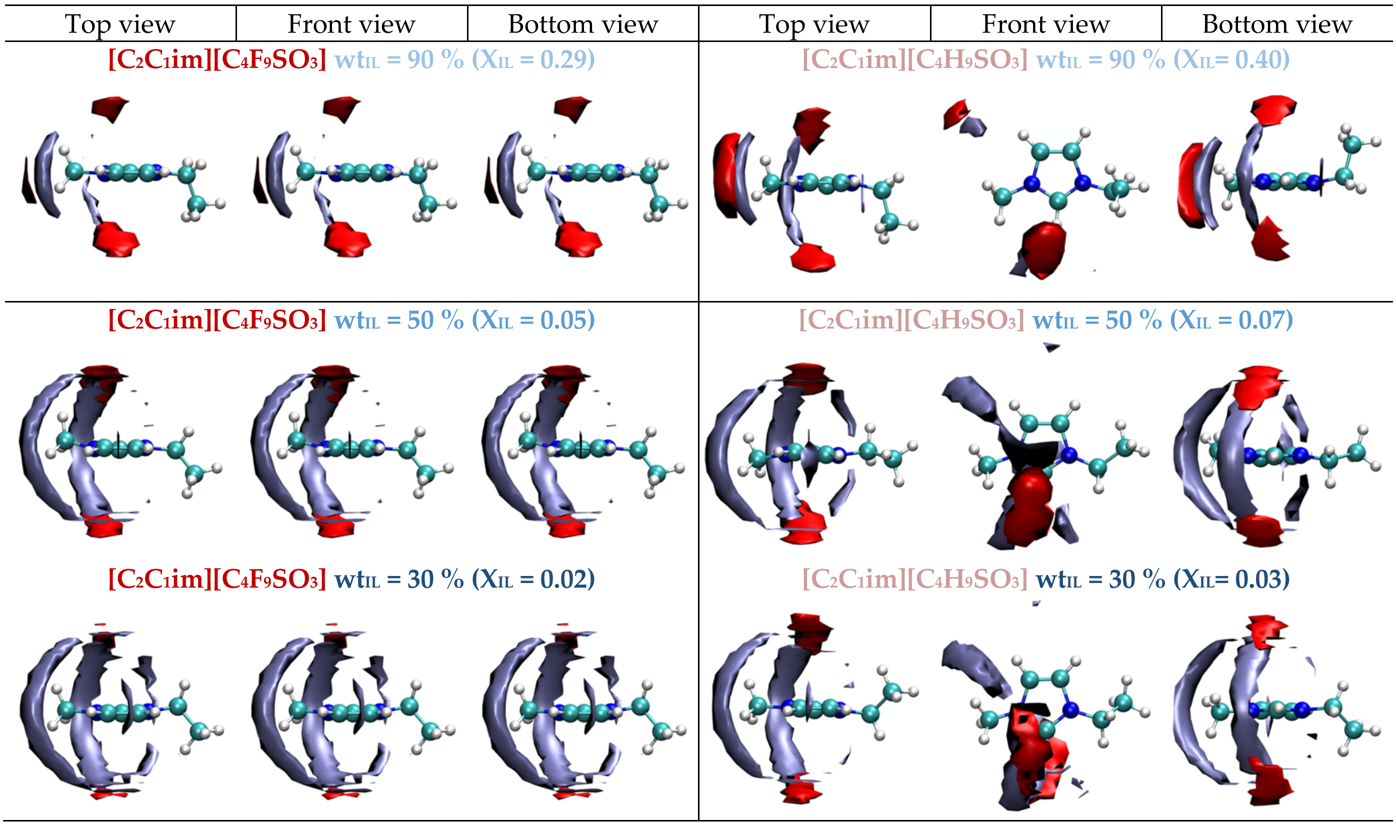

Figure S7. Selected Spatial Distribution Functions around the [C_2C_1im]$^+$ cation in aqueous mixtures of [C_2C_1im][$C_4F_9SO_3$] and [C_2C_1im][$C_4H_9SO_3$] at 300 K. The red and and light blue colours represent S (-$\underline{S}O_3$) atom of anion and water O atom, respectively. The isosurface value is 70% of the maximum number density.

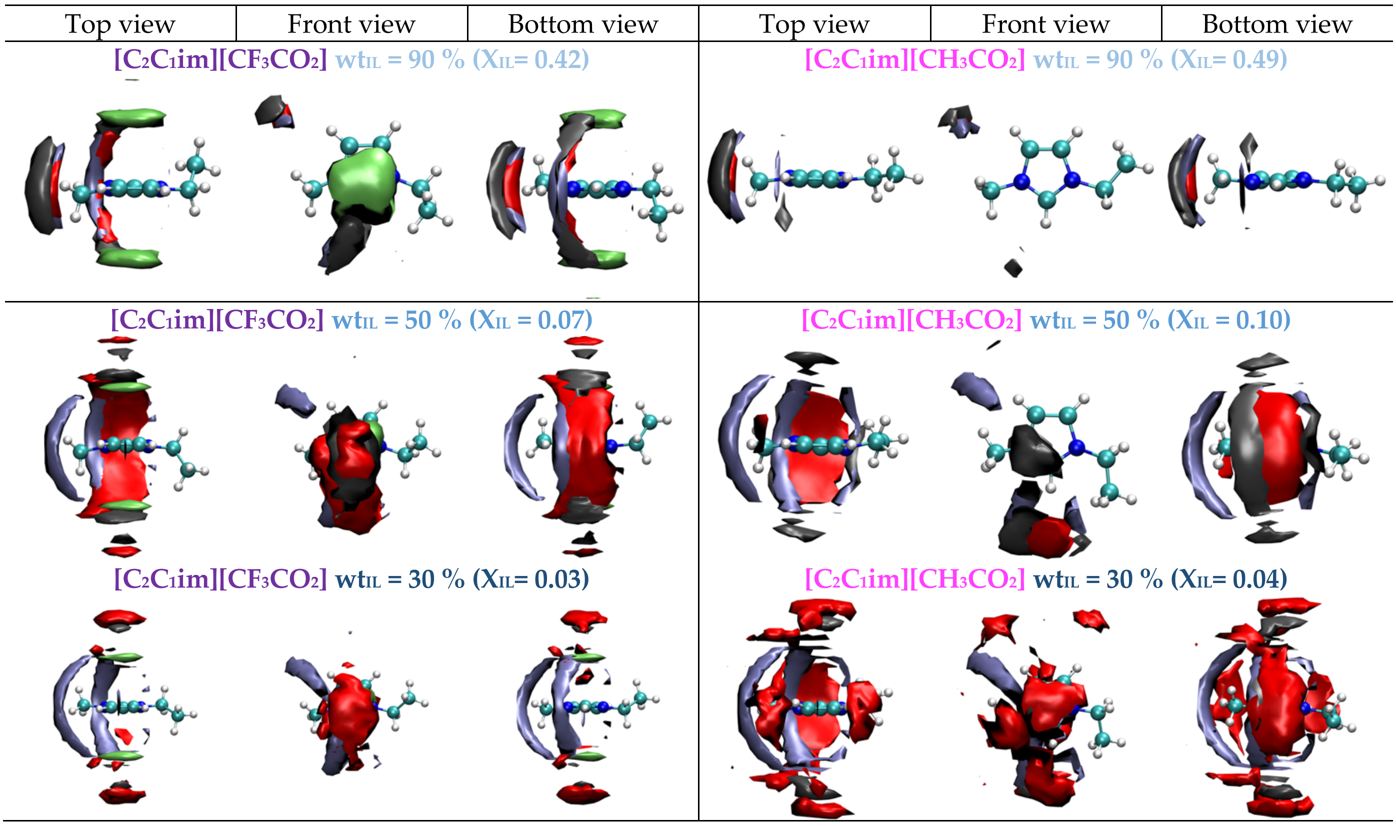

Figure S8. Selected Spatial Distribution Functions around the [C_2C_1im]$^+$ cation in aqueous mixtures of [C_2C_1im][CF_3CO_2] and [C_2C_1im][CH_3CO_2] at 300 K. The grey, red and green colours represent C, O (-C<u>O</u>$_2$) and F atoms of anion, respectively, and light blue depicts water O atom. The isosurface value is 70% of the maximum number density.

Top view	Front view	Bottom view

[C_2C_1im][$C_8F_{17}SO_3$] wt$_{IL}$ = 90 % (X_{IL} = 0.23)

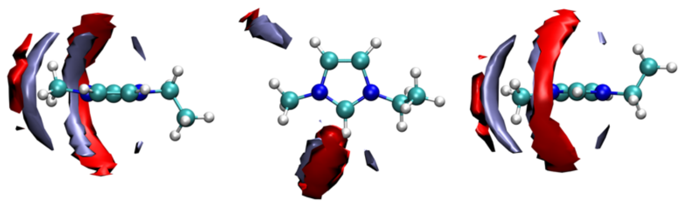

[C_2C_1im][$C_8F_{17}SO_3$] wt$_{IL}$ = 50 % (X_{IL} = 0.05)

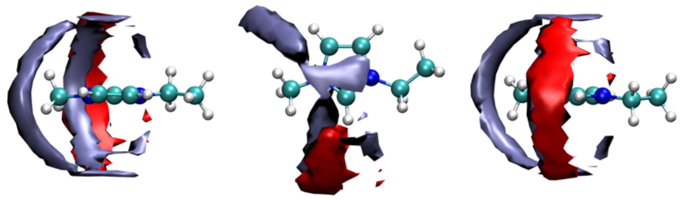

[C_2C_1im][$C_8F_{17}SO_3$] wt$_{IL}$ = 30 % (X_{IL} = 0.01)

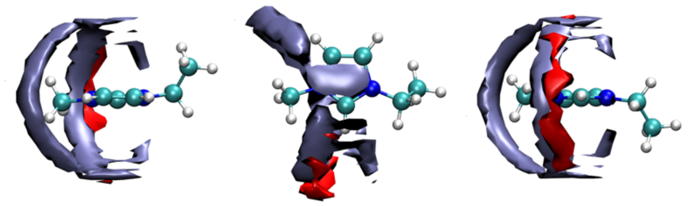

Figure S9. Selected Spatial Distribution Functions around the [C_2C_1im]$^+$ cation in aqueous mixtures of [C_2C_1im][$C_8F_{17}SO_3$] at 300 K. The red colour represent S (-$\underline{S}O_3$) atom of anion and light blue depicts water O atom. The isosurface value is 70% of the maximum number density.

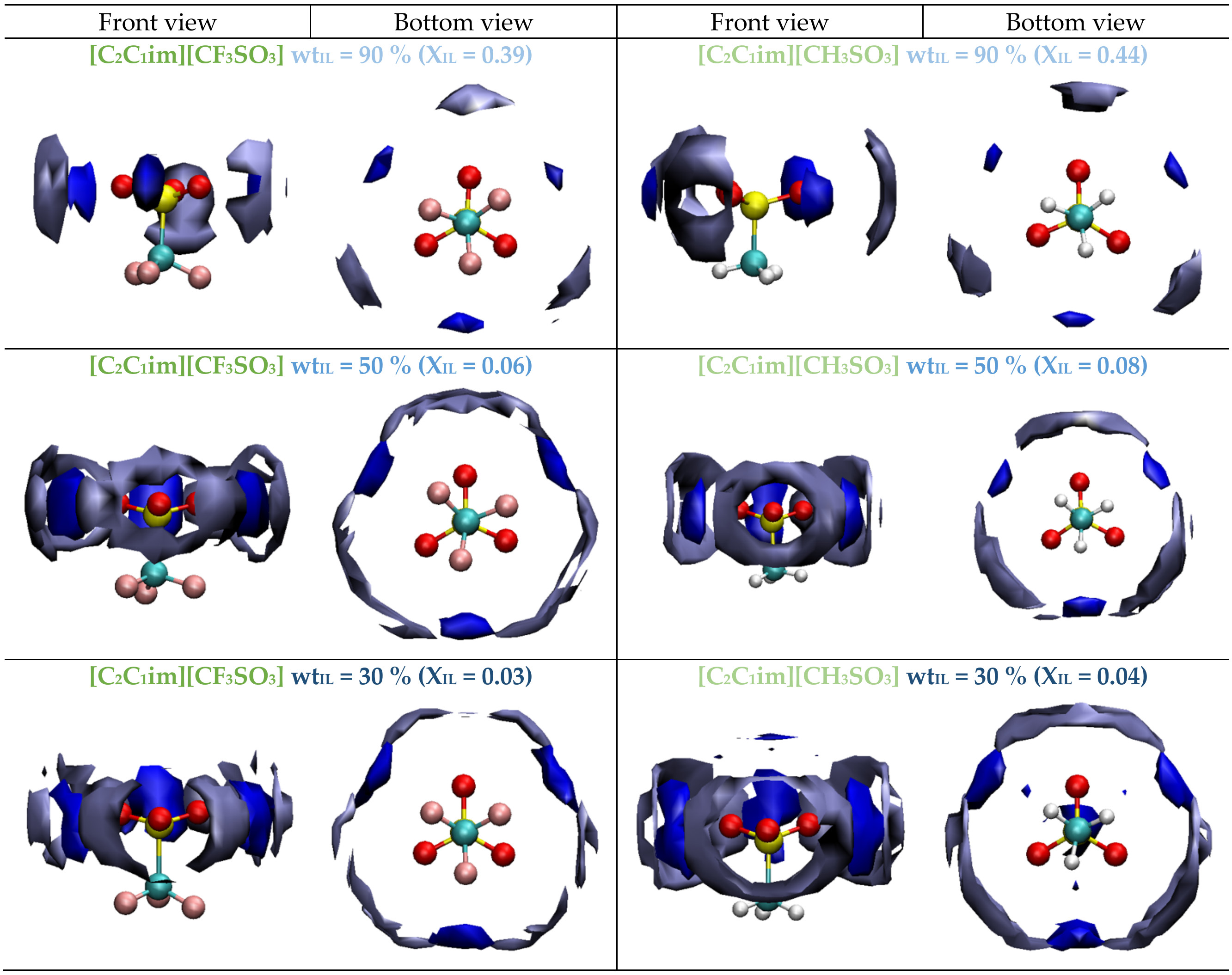

Figure S10. Selected Spatial Distribution Functions around the $[CF_3SO_3]^-$ and $[CH_3SO_3]^-$ anions in aqueous mixtures of $[C_2C_1im][CF_3SO_3]$ and $[C_2C_1im][CH_3SO_3]$ at 300 K. The blue colour represents H atom of cations attached to the carbon between nitrogen atoms of the imidazolium ring and light blue depicts water O atom. The isosurface value is 70% of the maximum number density.

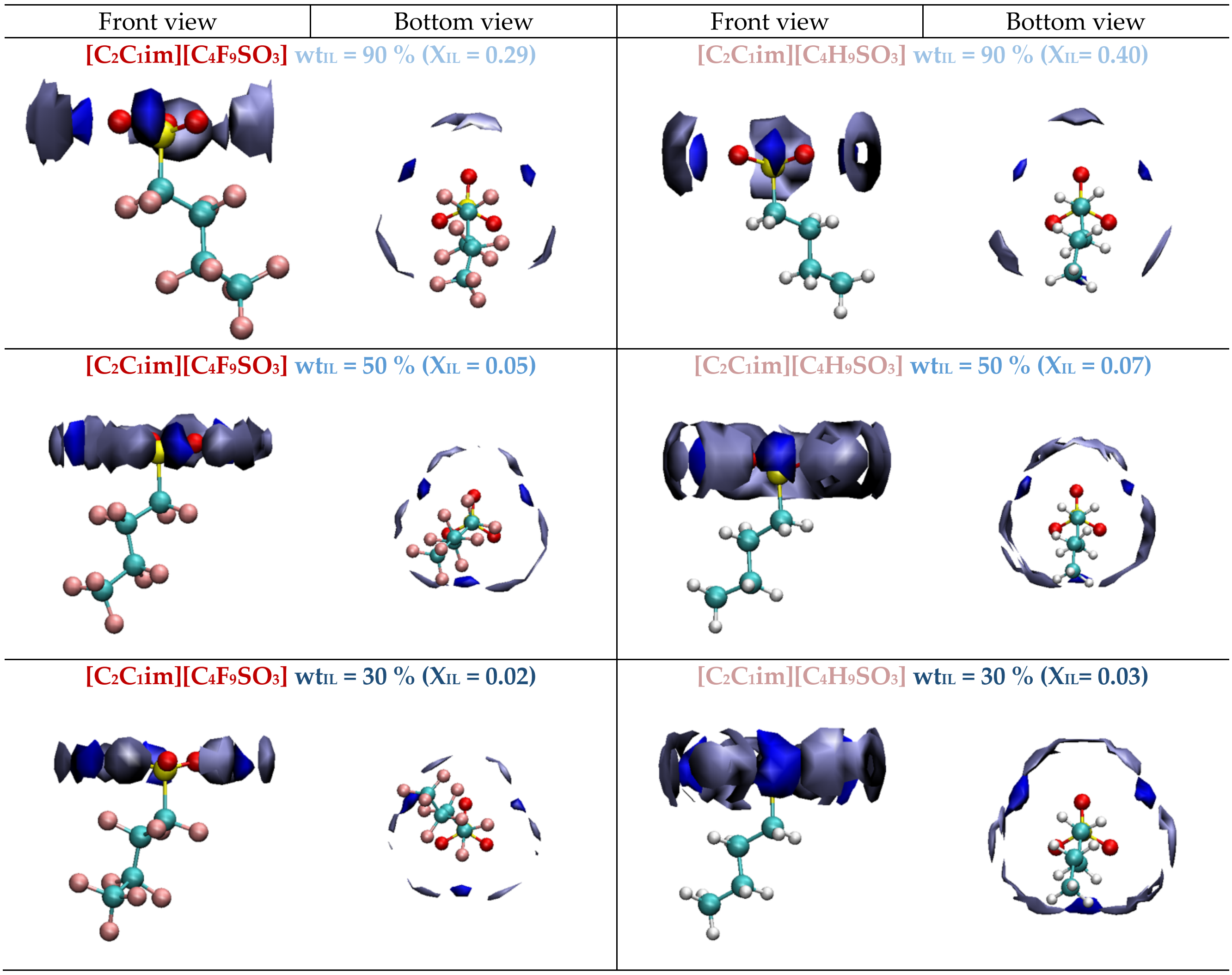

Figure S11. Selected Spatial Distribution Functions around the [$C_4F_9SO_3$]$^-$ and [$C_4H_9SO_3$]$^-$ anions in aqueous mixtures of [C_2C_1im][$C_4F_9SO_3$] and [C_2C_1im][$C_4H_9SO_3$] at 300 K. The blue colour represents H atom of cations attached to the carbon between nitrogen atoms of the imidazolium ring and light blue depicts water O atom. The isosurface value is 70% of the maximum number density.

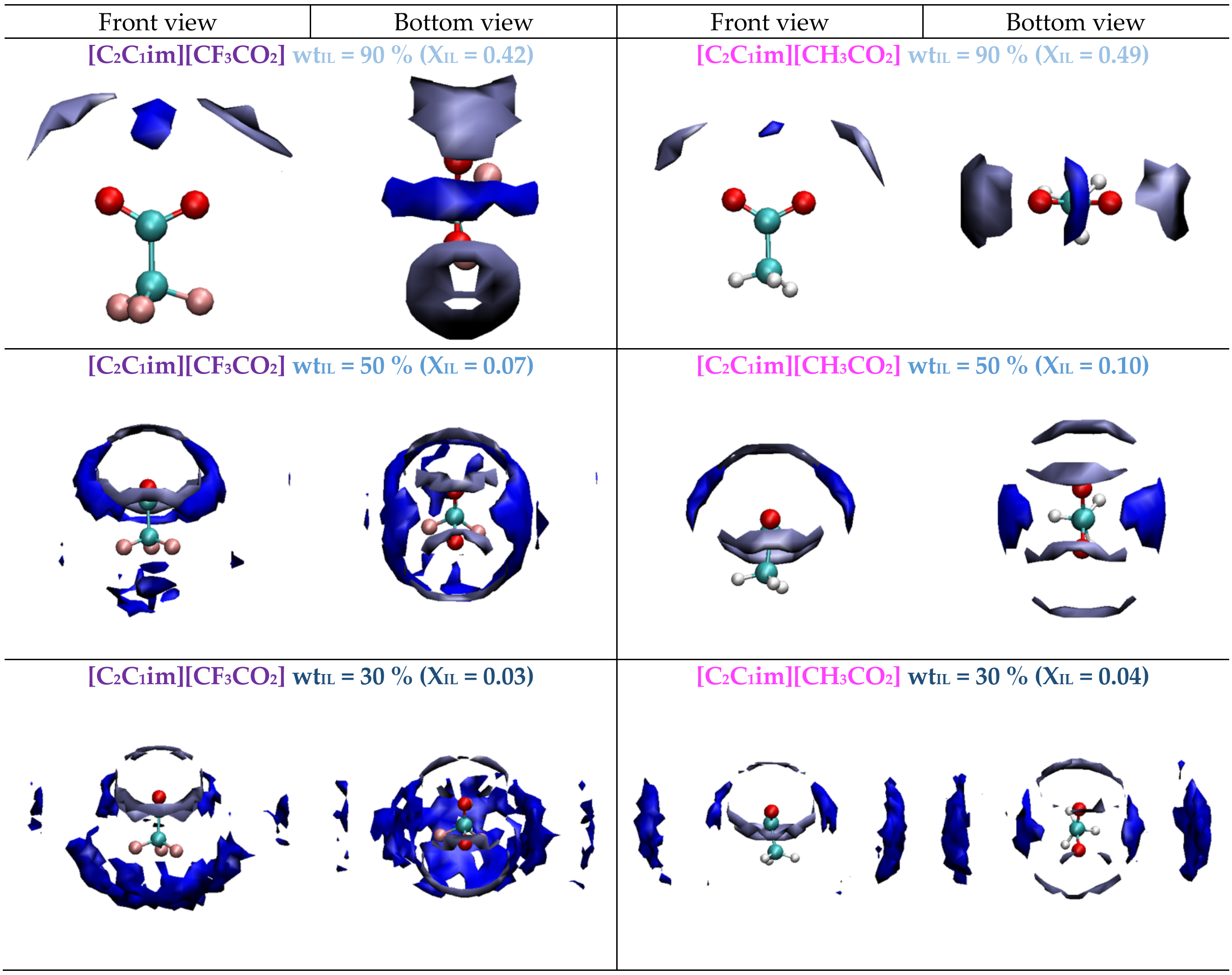

Figure S12. Selected Spatial Distribution Functions around the $[CF_3CO_2]^-$ and $[CH_3CO_2]^-$ anions in aqueous mixtures of $[C_2C_1im][CF_3CO_2]$ and $[C_2C_1im][CH_3CO_2]$ at 300 K. The blue colour represents H atom of cations attached to the carbon between nitrogen atoms of the imidazolium ring and light blue depicts water O atom. The isosurface value is 70% of the maximum number density.

Front view	Bottom view
**$[C_2C_1im][C_8F_{17}SO_3]$ wt_{IL} = 90 % (X_{IL} = 0.23)**	
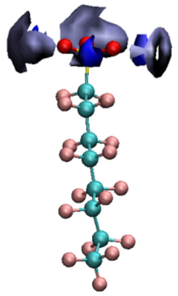	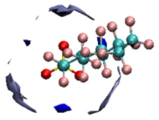
**$[C_2C_1im][C_8F_{17}SO_3]$ wt_{IL} = 50 % (X_{IL} = 0.05)**	
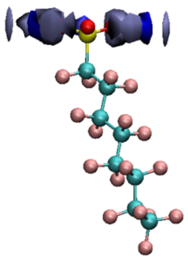	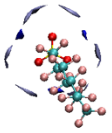
**$[C_2C_1im][C_8F_{17}SO_3]$ wt_{IL} = 30 % (X_{IL} = 0.01)**	
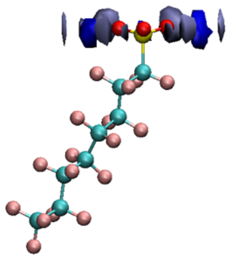	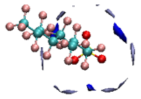

Figure S13. Selected Spatial Distribution Functions around the $[C_8F_{17}SO_3]^-$ anion in aqueous mixtures of $[C_2C_1im][C_8F_{17}SO_3]$ at 300 K. The blue colour represents H atom of cations attached to the carbon between nitrogen atoms of the imidazolium ring and light blue depicts water O atom. The isosurface value is 70% of the maximum number density.

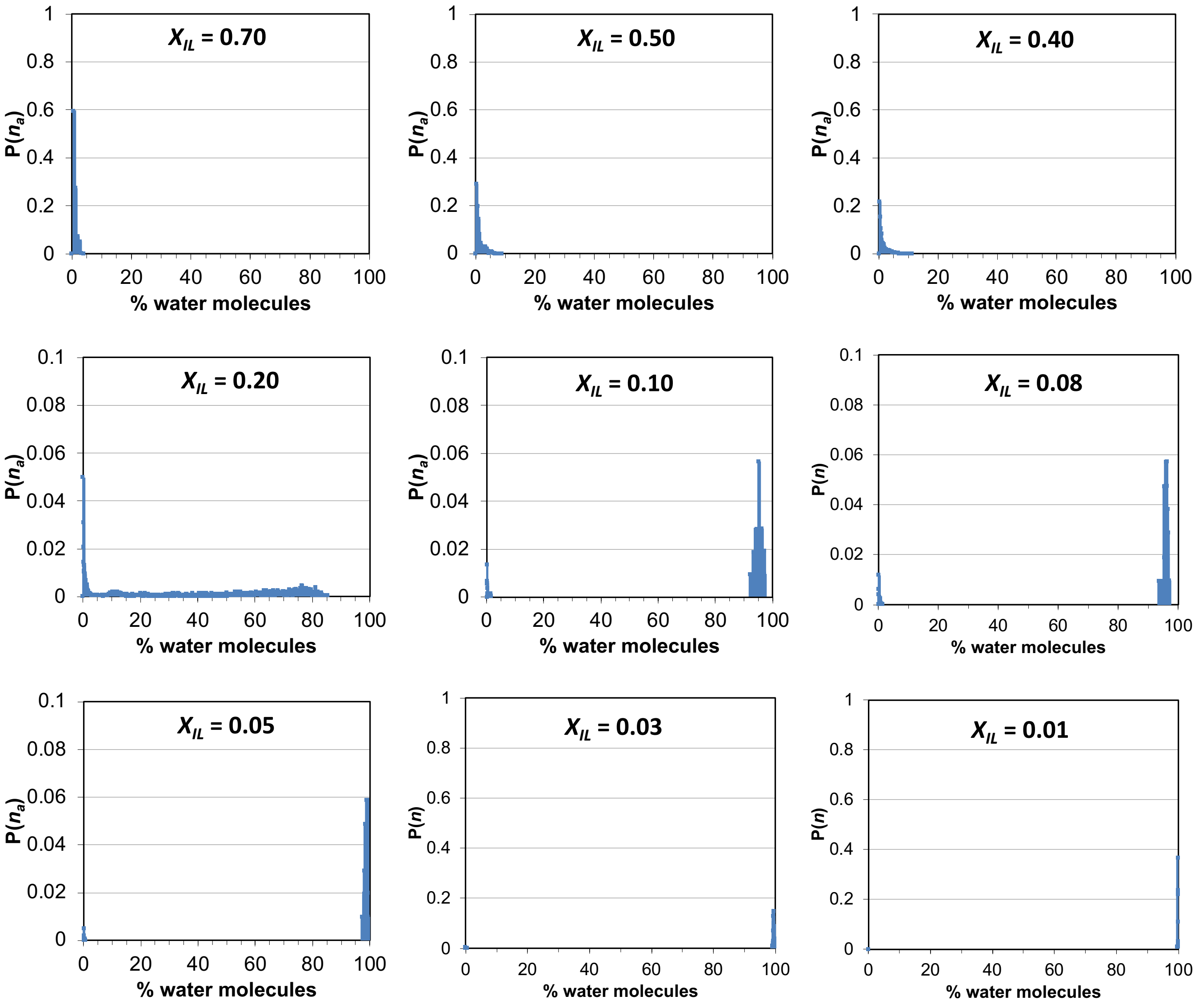

Figure S14. Discrete probability distribution of aggregate sizes, $P(n_a)$, as a function of aggregate size number, n_a, for water-water aggregates found in aqueous solutions of $[N_{1\ 2OH\ 2OH\ 2OH}][NTf_2]$ at 300 K.
